# Seed dispersal by vertebrates promotes invasion risk in the southern African grassland biome

**DOI:** 10.1007/s10661-025-14569-3

**Published:** 2025-09-19

**Authors:** L. R. Vukeya, T. M. Mokotjomela, N. Pillay

**Affiliations:** 1 School of Animal, Plant and Environmental Sciences, University of Witwatersrand, Private Bag 3, Wits 2050, Johannesburg, South Africa; 2 Directorate on National Botanical Gardens, Free State National Botanical Garden, South Africa National Biodiversity Institute, Rayton, Dan Pienaar, Danhof, P.O. Box 29036, Bloemfontein, 9310 Free State South Africa; 3 Center for Invasion Biology, School of Life Sciences, University of KwaZulu-Natal, Pietermaritzburg, 3200 South Africa

**Keywords:** Biodiversity, Non-native plants, Planning, Policy, Resource management

## Abstract

Vertebrate-mediated seed dispersal plays a crucial role in enabling the spread of invasive alien plants that threaten biodiversity in ecosystems such as the southern African grassland biome. Understanding the dynamics of the seed dispersal process by animals is important for assessing invasion risk and designing management interventions. This study investigated whether the southern African grassland biome is vulnerable to future plant invasions facilitated by frugivorous vertebrates in two known plant invasion hotspots in the Free State province: Boshof, dominated by *Opuntia engelmannii* (Cactaceae), and Fouriesburg, invaded by *Pyracantha angustifolia* (Rosaceae). We (1) monitored, documented, and compared the diversity of dispersal vector species in sites invaded by non-native woody and cactus species with non-invaded sites; (2) quantified the seed rain from vertebrates’ faecal samples and classified seeds and disperser species into functional groups to identify drivers of plant invasion of the grassland biome; and (3) assessed the effectiveness of vertebrate-mediated seed dispersal by measuring their post-ingestion viability. We predicted that frugivorous vertebrates are key drivers of fleshy-fruited plant invasions in this biome. We found taxonomically diverse dispersers (i.e. 27 families), with Bovidae and Numididae being dominant. Overall vector species diversity was high (*H* > 3.0), assuring effective population recruitment, although moderate in invaded sites (*H* > 2.5), and lower in non-invaded sites (*H* < 2.5). Over 9 months (March–November 2024), mammals dominated dispersal services (74%; *N* = 328 visits), followed by birds (26%), supporting our study prediction. We noted a high seed rain (i.e. 107,031 seeds) collected from 11,295 faecal samples associated with 43 vertebrate species. Invaded sites experienced significantly higher seed rain than non-invaded sites (*P* < 0.001) and were dominantly comprised of non-native species. Many seeds were medium-sized (1–5 mm), typical for successful invaders, and had a hard seed coat. Germination trials showed that ingested *P. angustifolia* seeds retained viability (46.7 ± 8.61%), similar to de-pulped controls (*P* = 0.53), while *O. engelmannii* seeds significantly lost viability (*P* = 0.002). The predicted seed dispersal distances were greater than 15.7 km for birds and ranged between 1.7 km and 8.6 km for mammals. We concluded that the grassland biome is vulnerable to further invasion by non-native plants dispersed by vertebrates, coupled with their long dispersal distances, and that integrating management of dispersal pathways into control plans is essential to optimise control efforts.

**Supplementary Information:**

The online version contains supplementary material available at 10.1007/s10661-025-14569-3.

## Introduction

Non-native plant invasions have a negative impact on local biodiversity (Yapi et al., 2018; Le Maitre et al., 2020; O’Connor & van Wilgen, 2020; IPBES, 2023) and livelihoods in rural areas and farms of South Africa (Mokotjomela et al., 2024a; Reynolds et al., 2020). Non-native plant invasions alter animal behaviour (Stewart et al., 2021; Ruland & Jeschke, 2020; Clusella-Trullas and Garcia, 2017; Dutra et al., 2011), modify vegetation structure (Adams et al., 2022; Mokotjomela et al., 2024a; Vukeya et al., 2025), and thus change the overall dynamics of the ecosystem’s functionality (Brooks et al., 2004; O’Connor & van Wilgen, 2020). They also alter food availability regimes, forcing vertebrates to shift their resource use, often to the detriment of indigenous species (Mokotjomela et al., 2013; Stewart et al., 2021). In the Mediterranean-climate region of South Africa, Mokotjomela et al. (2013) showed that birds prefer foraging on the abundant and nutritious fruits of the alien species bugweed *Solanum mauritianum* Scop. (Solanaceae) instead of native species, which significantly altered their seed dispersal services and the movement patterns of various vector species (Weber, 2002); Corbin & D’Antonio, 2010; Rogers & Chown, 2014; Gioria & Osborne, 2014; Divíšek et al., 2018; Mallick et al., 2019). Similar disruptions have been documented outside Africa. For instance, in the Great Basin region of North America, the invasion of cheatgrass *Bromus tectorum* L. (Poaceae) has created dense stands that physically inhibit the movement of native rodents and lizards, resulting in a decreased abundance and reduced richness of small vertebrate species (Rieder et al., 2010). Within southern Africa, comparable patterns have been observed. Coetzee et al. (2007) reported that the dense stands of *Acacia dealbata* have displaced native grasses and forbs, detrimentally affecting the richness and abundance of the Coleoptera community in the Drakensberg region of South Africa. In a North American example, the Busch Wildlife Conservation Area of Saint Charles, the invasion of the shrub Amur honeysuckle *Lonicera maackii* (Rupr.) *Maxim* (Caprifoliaceae) altered the behaviour of native seed-consuming mice (*Peromyscus* spp.) by providing them with refuge from predation (Dutra et al., 2011). Within the South African context, black wattle *Acacia mearnsii* and giant reed *Arundo donax* significantly alter patterns of the habitat used by carnivores and provide refuge for their prey (Hardesty-Moore et al., 2020; Boon et al., 2023). However, the intensity of the effect that non-native plants have on indigenous species is highly context-dependent (Mokotjomela et al., 2013), influenced by factors such as the specific characteristics of the invading species (Schaefer et al., 2008), the susceptibility of the invaded ecosystem (O’Connor & van Wilgen, 2020), and the dynamics of the resident species populations (Rejmanek et al., 2013).

Propagule pressure, combined with suitable environmental conditions and human-induced disturbances, has been attributed to the successful establishment and invasion of non-native plants (Le Roux et al., 2020; Simberloff, 2009; Lockwood et al., 2005). Propagule pressure entails a repeated influx of species’ reproductive parts from various dispersal vectors into a specific location and aids the invader to overcome biotic resistance to establish itself in new areas (Mack et al., 2000; Richardson & Thuiller, 2007). In support, Cassey et al. (2018) discovered that propagule pressure was consistently and positively linked to the successful establishment of non-native species. Dispersal distances also facilitate the invasion success through the formation of new populations that expand the occupancy of non-native species to the detriment of the native species (Milton et al., 2007; Sakai et al., 2001). The invasion intensity of black cherry *Prunus serotina* Ehrh. var. *serotina* (Rosaceae), for instance, is significantly driven by light and higher propagule pressure, with tree canopy cover reducing susceptibility to invasion in the Siemianice Experimental Forest in Poland (Jagodziński et al., 2019). Knowledge of the role of propagule pressure and seed dispersal patterns is crucial to managing the introduction of non-native species and their spread (Cassey et al., 2018; McGrannachan et al., 2021).

Seeds serve as the primary dispersal mechanism in many plant species, allowing them to colonise novel environments and to sustain their populations (Wang & Smith, 2002; Herera et al., 1994; Zhou et al., 2021; Kaushik et al., 2022). The nature of non-native plant seeds can significantly influence their ability to establish and invade new habitats (Gioria et al., 2014; Yu et al., 2024). Invasive plants often have suitable seed traits for invasion, such as small to medium-sized seeds, dispersed in large numbers and easily overcoming dormancy, allowing them to germinate rapidly and to out-compete indigenous species for vital resources (Rejmanek & Richardson, 1996; Núñez et al., 2017; Divíšek et al., 2018). The variations in the nature of the seed coat can also significantly impact water absorption and germination rates that determine germination (Zhang et al., 2020). For instance, thinner seed coats are associated with higher imbibition capacity and germination rates, while thicker coats can impede water absorption and reduce the germination percentage (Carrera‐Castaño et al., 2020; Zhang et al., 2020). Invasive species often possess distinct sets of characteristics that enable them to thrive in habitats where they are not naturally found (Divíšek et al., 2018; Sakai et al., 2001). Therefore, understanding these factors is crucial for developing adaptive management strategies for many non-native plant species (Gosper and Vivian‐Smith, 2009; Divíšek et al., 2018; Rayment et al., 2022).

Grassland habitats typically support few plant species that produce fleshy fruits (Canavan et al., 2022); however, the presence of numerous frugivorous birds and mammals facilitates the spread and establishment of favoured non-native fruit varieties (Mokototjomela et al., 2013; Adams et al., 2022). These fruits often have unique features such as extended availability, attractive colours, smaller and more numerous seeds, and higher nutritional content in respect of lipids, sugars, and higher water content (Galetti, 2002; Gosper et al., 2005; Traveset & Richardson, 2006; Theoharides and Dukes, 2007; Gosper & Vivian-Smith, 2009; Mallick et al., 2019; Sperry et al., 2021). One notable example is yellow firethorn *Pyracantha angustifolia* (Franch.) C.K. Schneid (Rosaceae), which is widespread in the protected areas, valuable grazing lands, and riverine habitats in the eastern Free State province of South Africa, and is dispersed by birds (Adams et al., 2022; Chari et al., 2020). Similarly*,* silverleaf cotoneaster *Cotoneaster pannosus* Franch. (Rosaceae) has encroached on montane grasslands of South Africa, specifically in the mountain regions and along watercourses, reducing plant diversity and altering ecosystem services (Canavan et al., 2022; Moloi et al., 2024). The success of these invaders can be partly attributed to their remarkable adaptability, which allows them to flourish even in environments that are typically unsuitable for native fleshy-fruited plants (van Kleunen et al., 2016).

The grassland biome in southern Africa is recognised as the most widely invaded vegetation type in South Africa (Table S2), ranking third after fynbos and savanna biomes (Van Wilgen & Wilson, 2018; O’Connor & van Wilgen, 2020; Zengeya and Wilson, 2023). Plant invaders include species that yield berries that are quickly spread in the biome by mammals (Adams et al., 2023; Canavan et al., 2022). Recent studies have shown that the grassland biome is vulnerable to plant invasions (Yapi et al., 2018; Henderson, 2019; O’Connor & van Wilgen, 2020; Bezeng et al., 2020; Vukeya et al., 2025). For example, Cactaceae species may aggressively transform natural habitats under global climate change (Masocha and Dube, 2018; Mokotjomela et al., 2024b). Species such as *Opuntia engelmannii* Salm-Dyck ex Engelm have had a devastating impact on the arid and semi-arid grasslands in South Africa and were declared an invasive species in 1984 (Henderson, 2019). It is commonly known as a small, round-leaved prickly pear and originates from North America (Henderson, 2020). In South Africa, it was introduced as a livestock fodder and for other horticultural purposes (Walters et al., 2011). The species is nationally categorised under the South African National Environmental Management: Biodiversity Act–Alien and Invasive Species Regulation (2020; NEM: BA-AIS) 1b, indicating that the species is widespread and has negative impacts on the economy, environment, and society and highlighting the urgent need for targeted eradication efforts (Department of Environmental Affair [DEF], (Department 2020); Kumschick et al., 2020). Yet, the species is a food resource for many vertebrates (Valero-Galvan et al., 2021).

Other plant invaders include the woody non-native species with devastating negative impacts on many ecosystems globally (Blignaut et al., 2010; Canavan et al., 2021; Pysek et al., 2012; Richardson et al., 2020; van Wilgen et al., 2022). Consistently, recent assessments suggest that the Rosaceae family has also been expanding its range with huge potential to transform many habitats through competitive interactions with native species (Chari et al., 2020; Richardson et al., 2020). For example, the Rosaceae have significantly invaded the eastern grassland biome, including *P. angustifolia* (native to China) and *C. pannosus* (native to China). *Pyracantha angustifolia* is an evergreen shrub that grows up to four metres tall and produces numerous attractive orange to red berries that are consumed by many grassland vertebrates (Fig. 3B; Adams et al., 2023; Bromilow, 2018; Henderson, 2020). These species were introduced into South Africa through the horticultural industry as ornamental plants and hedge growth owing to their ability to withstand cold temperatures (Chari et al., 2020; Canavan et al., 2022; Admas et al., 2023). All of them fall under NEM: BA category 1b (DFFE, 2020); they have particularly invaded the escarpment region of South Africa (Fig. 3A).

In a recent study, Vukeya et al. (2025) identified that the distribution of hotspots of non-native woody species has expanded within the grassland biome, concentrating in the eastern part of the biome, particularly in the KwaZulu-Natal and Gauteng provinces. Evidence for this observation is provided by the non-native cactus, Hudson Pear *Cylindropuntia pallida* rosea (DC.) Backeb. (Cactaceae), that is proliferating and displacing indigenous grassland vegetation in South Africa (Mokotjomela et al., 2024b). There is consensus that effective management should integrate species’ movement ecology in the response strategy, specifically distinguishing between the net effects of the natural and anthropogenic mechanisms (Mokotjomela et al., 2024a; Vukeya et al., 2025; Faulkner et al., 2025). However, comprehensive assessment reports—global (IPBES, 2023) and regional (Wilson et al., 2018)—suggest limited progress has been made to manage plant invasion threats effectively, partly due to a limited coherent understanding of the factors driving invasion and their impacts on the grassland biome, at least for this study.

This study aimed to investigate whether the southern African grassland biome hotspot is vulnerable to further plant invasions. We (1) monitored, documented, and compared the diversity of dispersal vector species in sites invaded by non-native woody and cactus species with non-invaded sites; (2) quantified the seed rain from vertebrates’ faecal samples, and classified seeds and disperser species into functional groups to identify drivers of plant invasion of the grassland biome; and (3) assessed the effectiveness of vertebrate-mediated seed dispersal by measuring their post-ingestion viability. We predicted that frugivorous vertebrates are the main drivers of fleshy-fruited plant invasion in the grassland biome and disperse large quantities of seeds with high-quality dispersal effectiveness. The value of this study lies in informing management actions aimed at reducing propagule pressure, targeting vertebrate dispersal networks, and preserving the indigenous biodiversity of grasslands.

## Method and materials

### Study sites

**Fig. 1:**
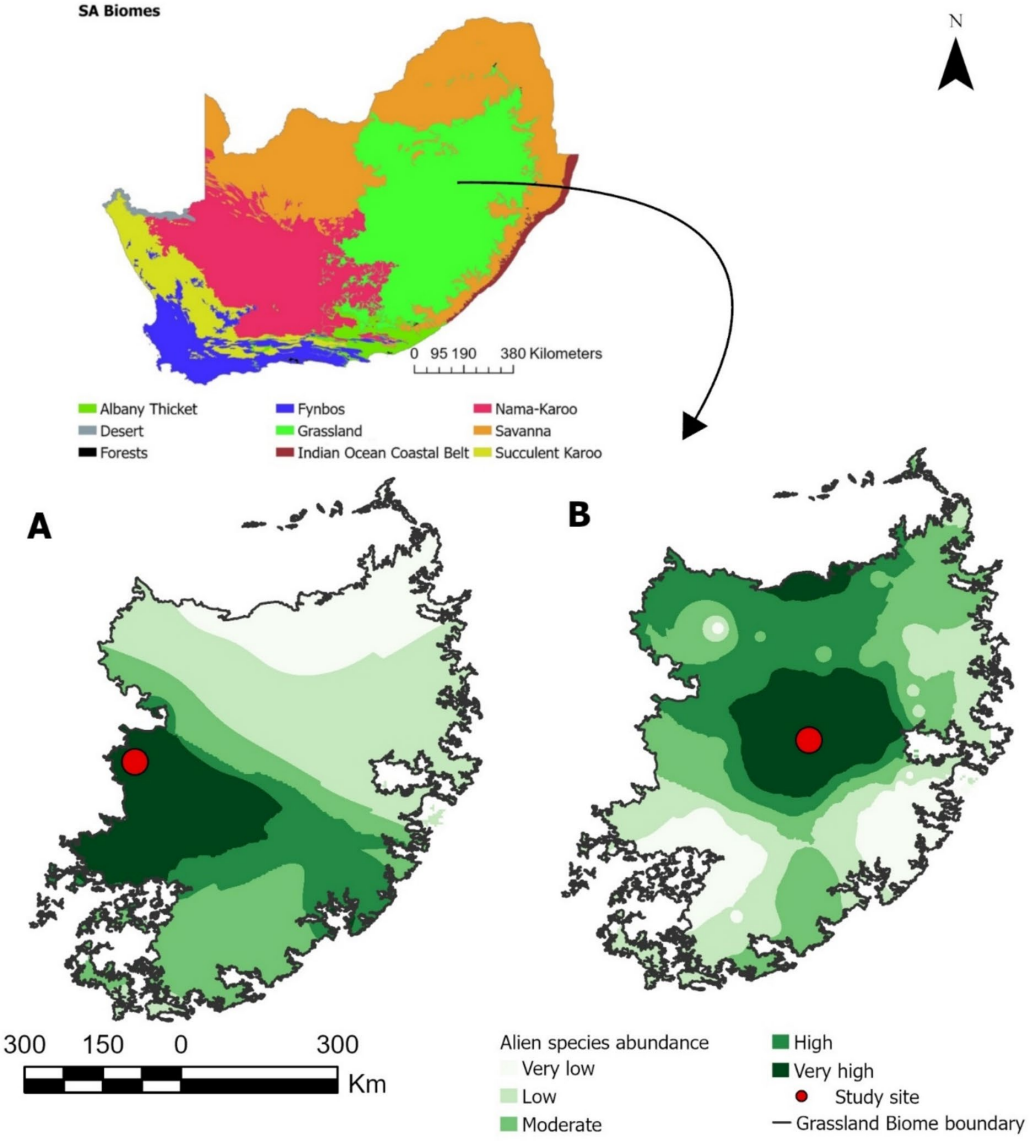
The study sites selected were situated within the grassland biome of South Africa (main map; Mucina and Rutherford, 2006). Smaller map A shows the distribution and abundance of *Opuntia engelmannii* in the grassland biome, with a red dot indicating the position of study site 1 at Boshof. Smaller map B shows the species distribution and abundance of *P. angustifolia*, with a red dot showing the location of study site 2 at Fouriesburg. The species abundance is displayed using various colours: dark green represents a hotspot, while pale light green is a coldspot––that is, the least abundant. The species occurrence data was gathered from several reputable platforms, including Global Biodiversity Information Facility (GBIF), iNaturalist, and the Southern African Plant Invader Atlas (SAPIA), ensuring a comprehensive and reliable set of records

**Fig. 2:**
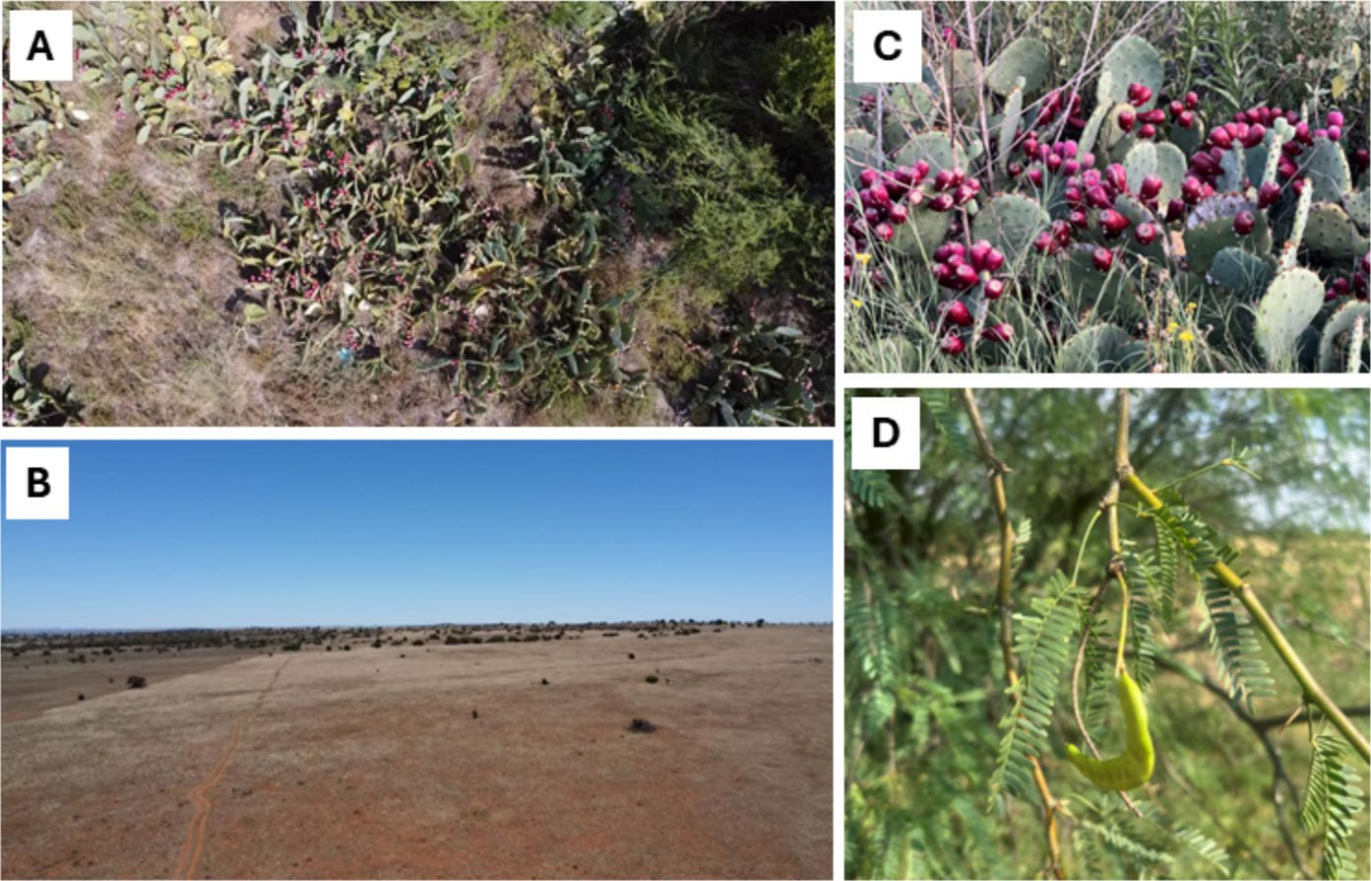
Study site 1. **A** Farm No. 432, located in the western Free State Clay Grassland at Boshof in the Free State province. **B** The non-invaded area is located near the R64 road, with native vegetation similar to the invaded area. **C** Small round-leaved prickly pear *Opuntia engelmannii* with ripened fruits. **D** Honey mesquite *N. glandulosa* pods that vertebrates could consume

**Fig. 3:**
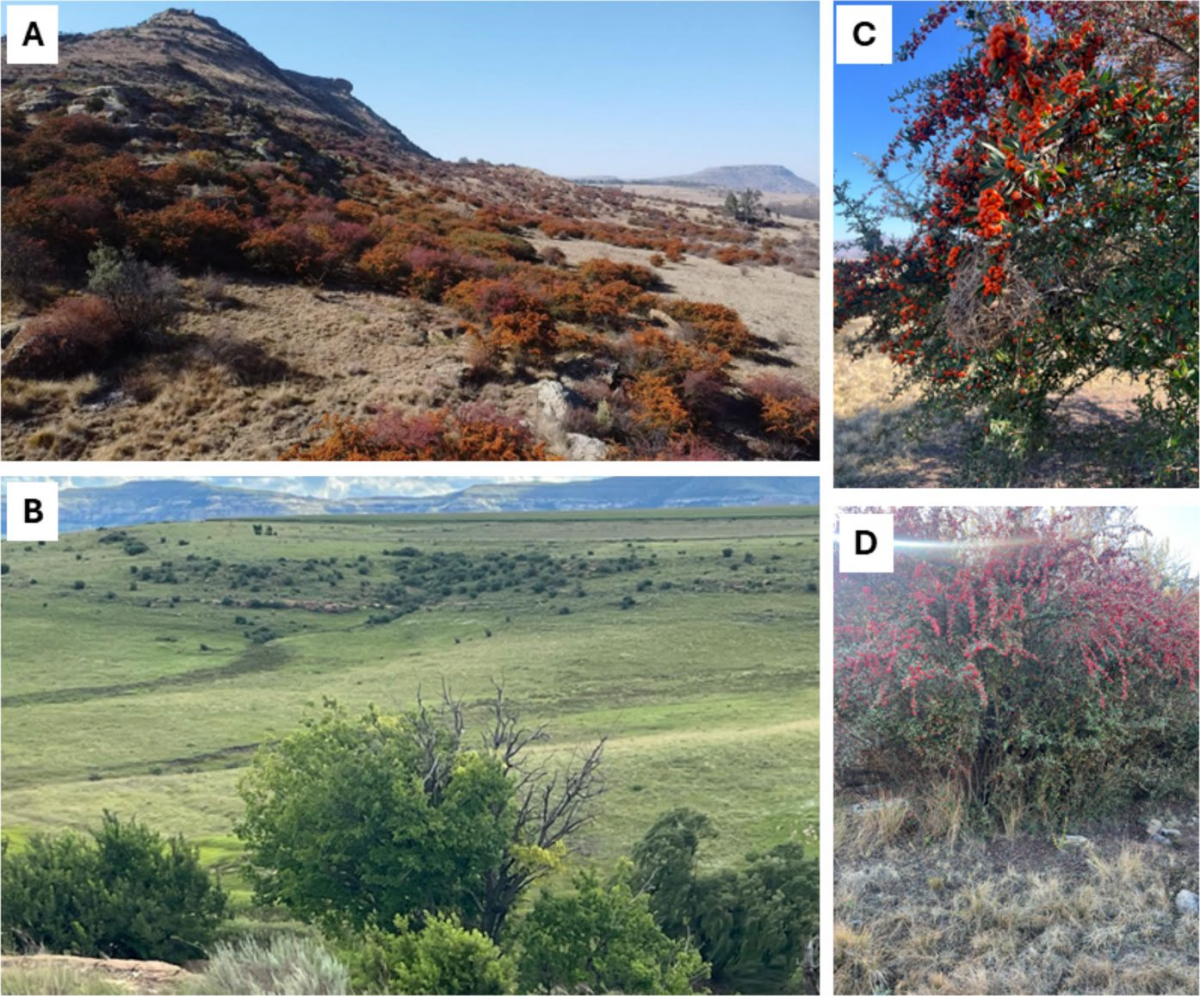
Study site 2. **A** Danskraal farm, located in the Eastern Free State Clay Grassland at Fouriesburg in Free State province, invaded by *Pyracantha angustifolia* and *Cotoneaster pannosus*. **B** The non-invaded area with indigenous vegetation similar to the invaded site. **C** Mature plants of *Pyracantha angustifolia*. **D** *Cotoneaster pannosus*, with ripe fruits that vertebrates could consume

The study was conducted in the central (Fouriesburg: 28° 40′ 52.63″ S, 28° 9′ 8.64″ E) and western (Boshof: 28° 32′ 12.23″ S, 25° 15′ 19.65″ E) regions of the southern African grassland biome from March 2024 to February 2025 (Fig. 1). Each study site comprised invaded and non-invaded areas. The selection of the invaded sites was based exclusively on the occurrence of fruiting woody and cactus invasive non-native plants, because these spread rapidly and transform grassland habitats (Masocha and Dube, 2018; Henderson, 2019; Canavan et al., 2022; Mokotjomela et al., 2024a), disrupting essential biome functions and ecosystem services (Le Matre et al., 2020; Moloi et al., 2024; Mokotjomela et al., 2024a). An elevated threshold of 1000 plant species distribution was deliberately selected, as it represents the most effective benchmark for assessing potential species distribution in the small study site (Valavi et al., 2022). This was quantified using imagery captured by a DJI Mini 4 Pro drone (Figs. 2A and 3A) (Dash et al., 2019; Rominger et al., 2021) to survey inaccessible habitats, to sample much larger areas quickly, reduce surface disturbance, and to measure demographic parameters for a greater number of individuals (Reckling et al., 2021; Rominger & Meyer, 2019; Rominger et al., 2021). The remote controller for the drone was interfaced to an iPhone 13 Pro. Flights were individually set up utilising the Map Pilot application designed for DJI, which was installed on the iPhone 13 Pro. The non-invaded areas were used as a control. The choice of non-invaded areas was based on vegetation types similar to those of the invaded areas and with limited land-use disturbance. Agricultural activities, including crop production and domestic livestock grazing, dominated land use at both study sites.

### Invaded site dominated by non-native cactus

The Boshof study site, situated in the western Free State Clay Grassland (Gh9, Dry Highveld Grassland bioregion) of the grassland biome in the southwestern area of Boshof town, within Free State province. (Figs. 1A and 2A; Mucina and Rutherford, 2006; Dayaram et al., 2019). The Boshof area receives approximately 450 mm of rainfall annually, mainly from November to March, with frequent frost in winter (Mucina and Rutherford, 2006). It has a mid-latitude steppe climate, with winter temperatures ranging from a low of 6 °C to a high of 31 °C during the summer (Mucina and Rutherford, 2006).

The Boshof site was invaded predominantly by fleshy-fruited cactus *Opuntia engelmannii* (family: Cactaceae). Other fruiting species at this site that are known to be consumed and dispersed by vertebrates include bushveld bluebush *Diospyros lycioides* Desf*.* (Ebenaceae), sweet thorn *Vachellia karroo* (Hayne) Banfi and Galasso (Fabaceae), karee *Searsia lancea* (L.f.) F.A. Barkley (Anacardiaceae), chinaberry tree *Melia azedarach L.*(Meliaceae), and honey mesquite *Neltuma glandulosa* Torr. var. torreyana (Fabaceae) (Gautier-Hion et al., 1985). The topography ranges from flat to undulating plains that are interrupted by dolerite sills (Mucina and Rutherford, 2006).

The selected non-invaded experimental control site had a similar native vegetation type to that invaded by *Opuntia engelmannii*––that is, the Western Free State Clay Grassland, which falls under the Dry Highveld Grassland bioregion located near the town of Boshof, beside the R64 (Fig. 2B). The vegetation is dominated by grass layers (Poaceae), with sparse dwarf woody species growing along riverbeds and hills, and similarly the non-invaded area was located approximately 5.7 km away from the invaded area.

### Invaded site dominated by non-native woody species

The study site in Fouriesburg was situated in the eastern Free State Clay Grassland (Gm4) of Mesic Highveld Grassland, about 10 km from the town of Fouriesburg in the eastern Free State province (Fig. 1b; Mucina and Rutherford, 2006; Dayaram et al., 2019). The Fouriesburg area receives rainfall ranging from 600 to 1000 mm annually, mainly from November to March, with occasional snow and frost in winter (Adam et al., 2023). Such areas undergo a harsh continental climate, experiencing winter temperatures that fall below 0 °C and summer temperatures that rise above 28 °C (Adam et al., 2023; Mucina and Rutherford, 2006). The site was predominantly invaded by fleshy-fruited members of the Rosaceae family, including *P. angustifolia* and *C. pannosus*.

The non-invaded experimental control site was also located in Fouriesburg, Free State province. It has a similar vegetation type to the invaded site––that is, Eastern Free State Clay Grassland (Fig. 3B). It is characterised by a diverse vegetation composition dominated by a grass layer with sparse woody shrubs, which provide refuge for various species (Mucina and Rutherford, 2006). The non-invaded area was located 7.5 km away from the invaded area to allow spatial independence of the samples (Hill et al., 2005).

### Documenting the fruit-eater species in the invaded and non-invaded areas

Three types of observational methods were used to document the presence of fruit-eaters: (1) direct observation; (2) indirect observation through identifying the faecal samples, tracks, and biological specimens or cues; and (3) camera trapping. The field data were collected three times at each study site (invaded and non-invaded) per season.

### Monitoring and documenting dispersal vectors

1. Direct observation
  Vertebrates directly sighted were recorded during the site visit. Furthermore, we identified observed species at a distance using binoculars (Bushnell 10 × 42 magnification). A digital camera (Canon models equipped with Sigma lenses ranging from 150 to 500 mm) was used to photograph various species for later identification.
2. Monitoring using camera traps
  Waterproof digital surveillance camera traps (Browning model no. BTC-8A) triggered by motion sensors were used to capture the presence of species that consume fruit (i.e. nocturnal and diurnal species) in the selected invaded and non-invaded areas during the study period. Ten camera traps were systematically deployed using a stratified random design (Kays et al., 2020) for 24 trials: 12 trials in the invaded area and 12 trials in the non-invaded area (replicated six times in each site, i.e. Boshof invaded site), with a single section being trapped for seven days consecutively. Cameras operated continuously, capturing data over 24-h cycles: 12 h during the day for diurnal activity and 12 h at night for nocturnal behaviour. Each motion trigger initiated a 60-s video recording in ultra-high resolution with a detection range of about 20 m. At night, the cameras used invisible infrared flashes on animals, ensuring that natural behaviour was not disrupted. Footage was downloaded after each sampling period. Repeated sightings of the same species were treated as a single observation within a short time frame. If there were collective nouns per sighting, they were classified as one per species. Species were identified using expert knowledge and validated with field guides (e.g. Apps et al., 2012) and online databases such as VMUS Vocalist.
3. Indirect observations––faecal samples, tracks, and biological specimens or cues
  Tracks, faecal samples, cues, and other observable signs of the presence of fruit eaters were identified and recorded using Indigenous knowledge during the site visits. For unidentified samples sighted in the field, digital images were captured with a scale reference for later identification using online platforms (https://vmus.adu.org.za/vmsplist) and field guides (Apps et al., 2012).

### Seed dispersal effectiveness of different vector agents

#### Seed collection from the faecal samples: quantity component

**Fig. 4:**
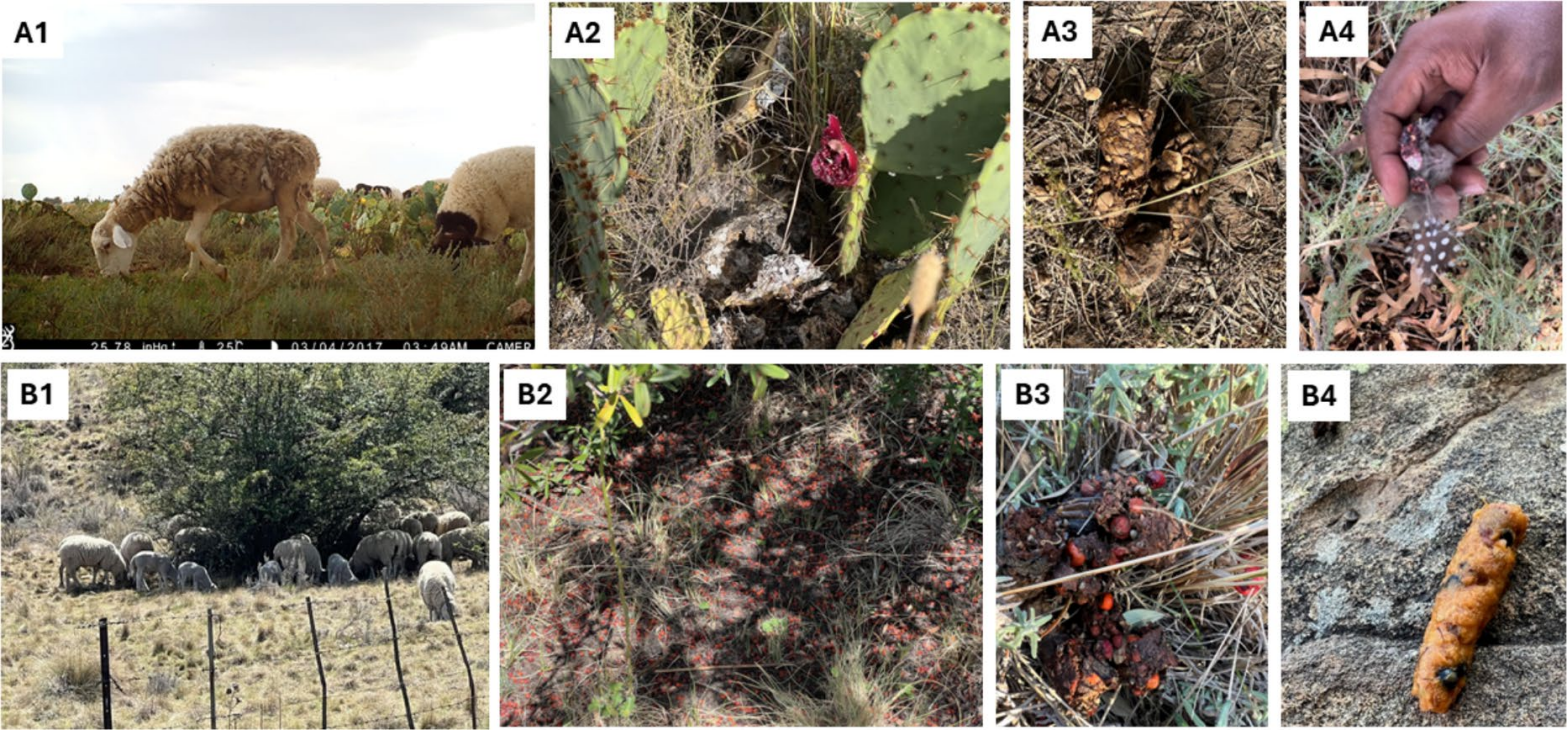
Field evidence from camera trap footage shows domestic sheep *Ovis aries* consuming and dispersing seeds of non-native plant species at the study sites (**A** Boshof and **B** Fouriesburg). *Opuntia engelmannii* fruits being consumed by *O. aries* (A1 and A2). Dropped fruits under the parent trees (B2) are consumed by *O. aries* (B1). Masticated seeds trapped in the faecal sample (A3, A4, B3, and B4)

To evaluate seed dispersal patterns, faecal samples (either fresh or dry) containing seeds of different species (Fig. 4) were collected in the walk transects. To avoid rehydration/dehydration, the collected faeces were stored in brown bags at the Free State National Botanical Garden seed store. The faecal samples were identified based on observable characteristics like colour, shape, texture, and size, as well as the use of a field guide (e.g. Carruthers, 2016). The samples were then gently crushed in the Free State National Botanical Garden potting shed using a wooden rod to expose their contents. This enabled the separation, counting, and recording of masticated seeds (identified by their seed coats) for each faecal sample. The seeds were identified using their morphological structure (size––length and width, coat, and shape) (Ulian et al., 2019; Vukeya et al., 2022), existing knowledge, various botanical reference books (e.g. Ulian et al., 2019), and online data such as the National Biodiversity database managed by the South African Biodiversity Institute (http://pza.sanbi.org). To identify tiny seeds, a compact × 100 metal mini science microscope was used (Song et al., 2018; Vukeya et al., 2022). The germination trial for unidentified seeds was used for possible identification, and when it was impossible to identify the seeds positively, plant species were labelled as “unknown1”. Faecal samples were collected in the invaded and non-invaded areas in both study sites to infer the potential risks of invasion in the future of the grassland.

To ascertain non-native species that are likely to invade the grassland and vulnerable biome units, the seeds in the faecal samples were classified into seed size by measuring their weight (using a SWAN series digital scale in grams) and length (using a digital calliper). Radny et al. (2018) and Rejmánek and Richardson (1996) indicated that non-native plant species with small seeds may endure competitive stress and germinate more quickly, with the potential to be more widely dispersed, and so support successful invasion more than large seeds do. Some seeds weighed too little for the fine-scale balance available––0.01 g. Therefore, we systematically selected 20 seeds and weighed them, replicating this process 20 times. The weight of the 20 seeds was divided by 20 to obtain the average weight of a single seed. Because moisture can confound seed weight (Hay et al., 2023), the length of each seed was measured with a Digital Vernier Calliper (i.e. the longitudinal dimension of each seed) as a surrogate of size and replicated 20 times for each plant species. Only plant species that had more than 20 seeds were considered. To categorise the size, we used the categories described by Yang et al. (2025): tiny seeds (less than 1 mm), medium seeds (1–5 mm), and large seeds greater than 5 mm). The seed coat thickness plays a crucial role in the success of invasion by non-native species because it can impact germination success. Therefore, we classified the seeds according to their texture by squeezing and feeling them between two fingers (i.e. thin, soft, or hard).

#### Quality component: seed viability and germination

The seed viability test of the dominant species was conducted using germination trials in a Quonset-type greenhouse at the Free State National Botanical Garden from October 2024 to April 2025. A pre-sowing sterilisation protocol was used for the germination trays, involving a bleach and water solution to manage the proliferation of fungi and insect pests (Nichols, 2005; Vukeya et al., 2021). A total of 10 intact (collected from the faeces) seeds per tray were planted, with each treatment replicated 10 times. Manually de-pulped seeds were used as a control for gut processes by the vertebrates, and a total of 10 seeds were sown per tray and replicated 10 times. For the de-pulping process, seeds were separated from the surrounding fleshy pulp by squeezing the fruit to release the seed between two fingers. The potting mixture used for sowing the seeds was a well-drained organic medium, and the trays were placed in the greenhouse, with watering provided as required in the mornings and late afternoons.

#### Role of the vectors in seed dispersal distances

The attributes (i.e. adult body mass) of the observed dispersal vectors (i.e. mammals and birds) were used to determine the potential seed dispersal distances using allometric models. The adult body mass (BM) was used to predict gut retention time (GRT) in hours for ingested seeds, as well as movement capacity (MC) in kilometres for potential dispersal range. This approach aligns with previous studies (Jordano, 2000; Schurr et al., 2009; Tsoar et al., 2011; Mokotjomela et al., 2015; Vukeya et al., 2023). The data on bird species sizes were obtained from Hockey et al. (2005), while we used the mammal categories described in Alemu et al. (2024). The product of the GRT and flight speed for birds and home range for mammals (i.e. MC) was considered as the potential seed dispersal distance (Mokotjomela et al., 2013; Msweli, 2021; Schurr et al., 2009).

For bird species, the equations from Robbins (1993) and Calder (1996) were used to estimate the relationship:

1 $${\text{GRT}}_{\left(\text{b}\right)}={1.6 \{\text{BM}\}}^{0:33\text{kg}}$$

(1.6 and 0.33 are allometric constants; subscript “b” denotes birds), and

2 $${\text{MC}}_{\left(\text{b}\right)}={15.7 \{\text{BM}\}}^{0:17\text{kg}}$$

(15.7 and 0.17 are allometric constants; subscript “b” denotes birds).

For mammal species, the GRT (in hours) was estimated using the allometric equation adopted from Steuer et al. (2011).

3 $${\text{GRT}}_{\left(\text{m}\right)}={31.0 \{\text{BM}\}}^{0:01}$$

(31.0 and 0.01 are allometric constants; subscript “m” denotes mammals).

The MC (measured in kilometres) was determined utilising equations described in du Toit (1990).

4 $${\text{MC}}_{\left(\text{m}\right)}={0.024 \{\text{BM}\}}^{0:18}$$

(0.024 and 0.18 are allometric constants; subscript “m” denotes mammals).

### Data analysis

We combined the data of the documented fruit-eating species in each invaded and non-invaded component to assess the overall frequency of foraging visits by species in the two sites. To ascertain the vector species diversity and whether the studied sites (invaded and non-invaded) differed, we computed the Shannon–Wiener diversity index (SWDI) using the documented animal foraging frequencies.

To identify the most suitable model during data analysis, the data was assessed for over-dispersion or under-dispersion by checking the variance or deviance value, which should be approximately 1 in the output from the Statistical Package for the Social Sciences (SPSS) software (version 29, IBM, New York, NY, USA).

A generalised linear model (GLM) applied to the data was dismissed if the variance was notably higher or lower than 1. The Omnibus tests of the model coefficients, along with a likelihood ratio chi-square test showing strong significance, were utilised to ensure that the findings could be interpreted with confidence. Ultimately, the model that exhibited a relatively lower variance was chosen as the best-fitting model for the data.

The count data for mammals and birds were analysed for within- and between-site differences using a GLM fitted with a Poisson distribution and log link. Animal counts were the response variable, while the type of vertebrates (i.e. mammals and birds) was a predictor variable nested within sites.

The overall quantity of dispersed seeds was estimated as the average total number of seeds identified in all faecal samples and the total average foraging frequency of all animals. The daily rate of seed dispersal was generated by dividing the estimated total average seed count for all animals by the total number of days spent camera-trapping in the field. A GLM with a negative binomial error distribution was used to analyse differences in the number of fruits/seeds defecated by vertebrates (i.e. mammals and birds) per study site. The number of seeds per species was the response variable, and vertebrate classes and study sites were the predictor variables. Tukey’s post hoc test was used to analyse the differences in seed counts between invaded and non-invaded areas. The plant species seeds’ functional groups (i.e. seed size and cover) were examined to identify seed attributes important for the grassland biome invasion. The GLM fitted with a negative binomial error distribution was used to analyse differences in seed types defecated by vertebrates in each study site. The seed count was the response variable, while seed size, the nature of the seed cover, and whether the seed was non-native or indigenous were predictor variables nested within sites.

The germination rates of de-pulped seeds, serving as our experimental control, were rigorously compared to those of seeds ingested by vertebrates, treated as our primary focus per plant species (mean ± SE%). This comparison highlights the significance of vertebrate interactions for seed germination outcomes. To analyse variations in the quantity of seeds that germinated in the treatments, a GLM with a negative binomial error distribution was used. In this model, the germination count was the response variable, while the types of treatment (i.e. vertebrate-ingested and de-pulped seeds) were identified as the predictor variables.

## Results

### Species diversity of dispersal vectors

We recorded 328 animals through direct and camera trap sightings, comprising 43 species dominated by mammals and birds (61% and 32%, respectively; *N* = 328; Table S1), spanning 27 families. Notably, many foraging species were classified as Bovidae and Numididae families (48% and 7.4%, respectively).

The Shannon–Wiener diversity indices showed a high level of overall species diversity (*H* = 3.01; Table 1). The invaded sites exhibited moderate species diversity, with Boshof (*H* = 2.52) and Fouriesburg (*H* = 2.97) displaying this characteristic. In contrast, the non-invaded sites showed lower species diversity in both study sites (*H* < 2.49).

**Table 1:** Shannon–Wiener diversity indices comparing the diversity of vector species across the two study sites

Diversity indices			*Boshof town*		*Fouriesburg town*	
		Overall species diversity	Non-invaded	Invaded area	Non-invaded	Invaded area
**Shannon–Wiener diversity indices**	***H***	3.01	1.82	2.52	2.32	2.82
	***H***_**max**_	3.76	1.95	3.09	2.48	3.43
	**Equitability**	0.79	0.94	0.81	0.93	0.81

Shannon–Wiener diversity indices H: very low––1.99 and below; low––2.0 to 2.49; moderate––2.50 to 2.99; high––3.00 to 3.49; and very high––3.50 and above (Fernando et al., 1998)

A notably larger quantity of mammals, compared to birds, visited and consumed fruits in the study plants at both study sites (Wald *χ*^2^ = 165.09; *df* = 41; *P* < 0.001). Among the identified species, the common duiker *Sylvicapra grimmia* showed the highest relative abundance (21%; *N* = 328), followed by domestic cattle *Bos taurus* (12.2%; *N* = 328).

**Fig. 5:**
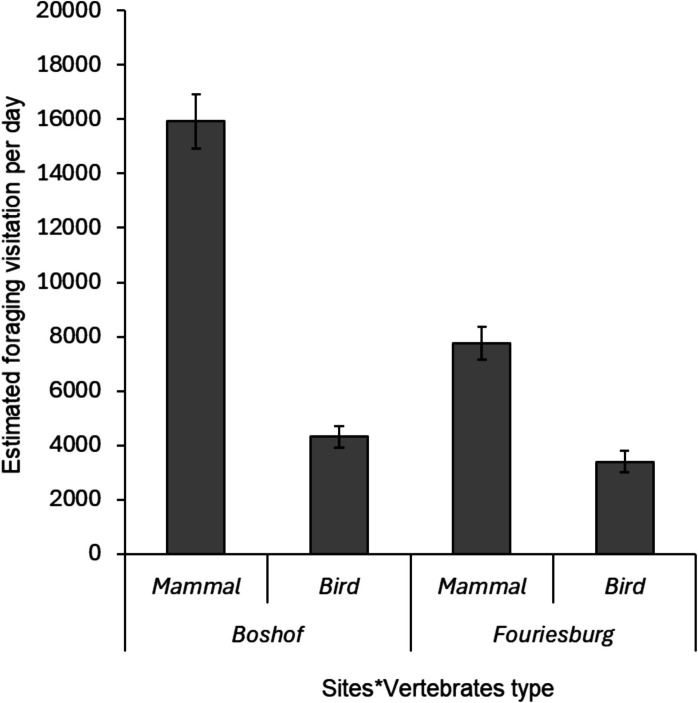
Foraging frequency of different vertebrates documented daily in the study sites

Overall, dispersed seeds in both study sites were significantly dominated by mammals (Wald *χ*^2^ = 973.79; *df* = 1; *P* < 0.001; 74%; Fig. 5), with large-sized mammals (i.e. *S. grimmia*, *Ovis aries* and *B. taurus*) predominating in Boshof (98.57%; *N* = 1880; Fig. 5), while medium-sized mammals (i.e. *Hystrix africaeaustralis* and *Cynictis penicillata*) were dominant in Fouriesburg (99.12%; *N* = 917; Fig. 5).

### Quantity component: number of seeds

**Fig. 6:**
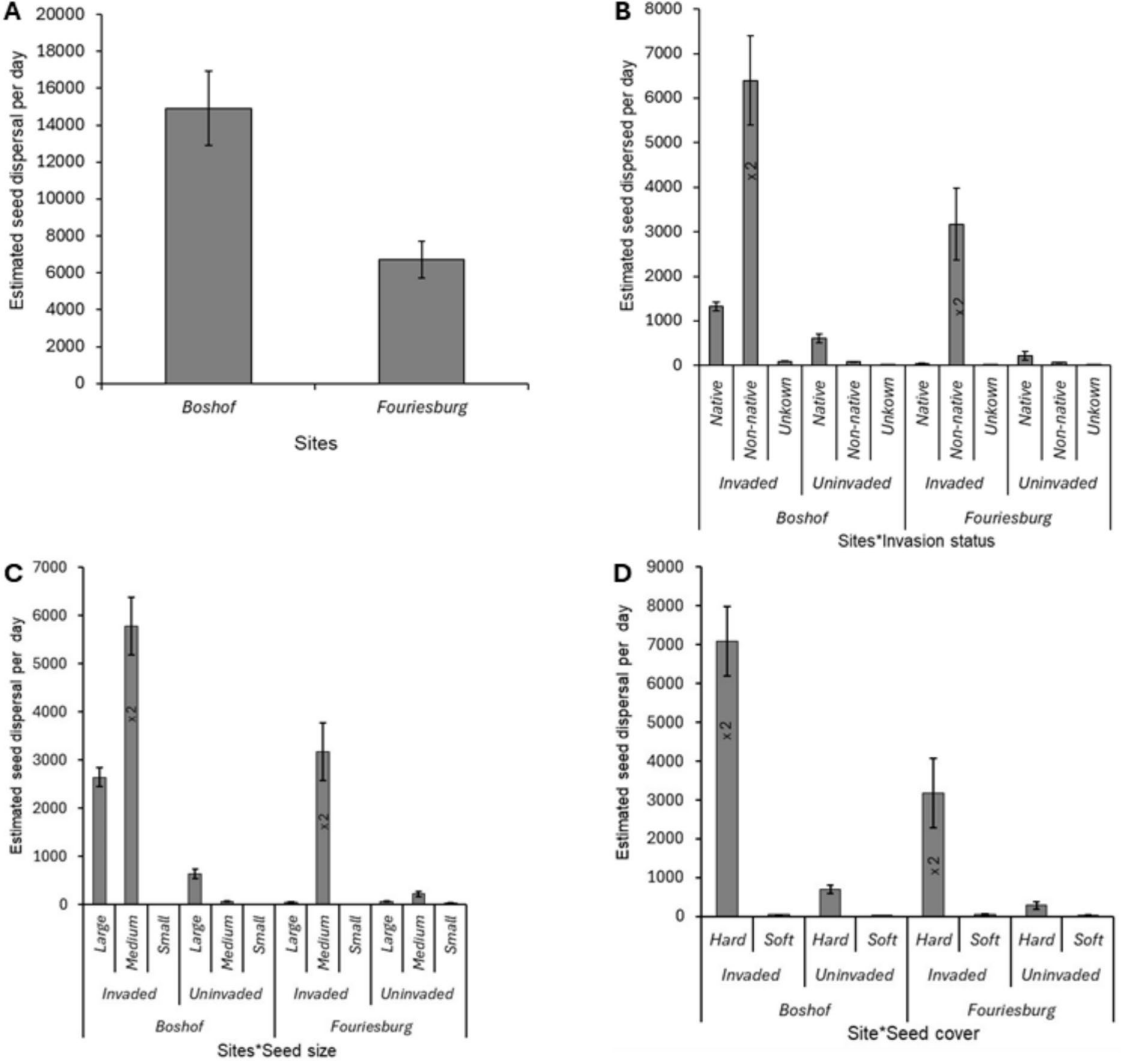
Seed rain and seed classification of the species using their functional traits. **A** The total number of seeds in each site. **B** Seed species of origin: non-native vs indigenous. **C** Seed size. **D** Seed cover texture

A total of 11,295 faecal samples containing 107,031 seeds in nine vertebrates (i.e. mammals and birds) were collected over nine months (March to November 2024) in the two study sites. Fruits/seeds of different plant species were consumed in significantly different numbers by the vertebrates in the grassland biome (Wald *χ*^2^ = 119.2; *df* = 11; *P* < 0.001; Fig. 6A). Tukey’s post hoc tests showed that invaded sites had significantly larger seeds dispersed than non-invaded areas (*P* < 0.001). Overall, the number of dispersed seeds between the indigenous species versus the non-native species differed significantly (Wald *χ*^2^ = 50.06; *df* = 2; *P* < 0.001; Fig. 6B). The non-native seed species comprised 89.3% (*N* = 107,031; Fig. 6B) of the total dispersed seeds. A significantly large number of seeds were classified as medium (1–5 mm) in size (Wald *χ*^2^ = 176.57; *df* = 2; *P* < 0.001; Fig. 6C) at both study sites (Boshof: 78.01%; *N* = 87,528 and Fouriesburg: 97.98%; *N* = 19,494; Fig. 6C). Also, a significantly larger number of seeds had a hard seed cover texture in the grassland biome (Wald *χ*^2^ = 4.49; *df* = 1; *P* = 0.034; Fig. 6D) (99.5%; *N* = 107,031), whereas the remaining seeds had a softer exterior cover in both sites (Fig. 6D).

### Quality component: seed viability

The ingested seeds of *P. angustifolia* had a viability of 46.7 ± 8.6% (*N* = 100; Table 2), while *Opuntia engelmannii* had a seed viability of 5.4 ± 0.8% (*N* = 100; Table 2). There was no notable distinction in germination rates between ingested and de-pulped seeds of *P. angustifolia* (Wald *χ*^2^ = 0.394; *df* = 1; *P* = 0.532; Table 2). In contrast, significantly more of the manually de-pulped seeds of *Opuntia engelmannii* germinated compared with the ingested seeds (Wald *χ*^2^ = 9.16; *df* = 1; *P* = 0.002; mean ± SE%: 15 ± 4.9; Table 2).

**Table 2:** Seed viability (%) of dominant fruit-bearing non-native plant species ingested and defecated by different dispersal vectors

Plant species	Family	Life form	Total dispersed seed germination (mean ± SE%)	
			Depulped seeds	Ingested seeds
*Opuntia engelmannii*	Cactaceae	Succulent	15.0 ± 4.9	5.4 ± 0.8
*Pyracantha angustifolia*	Rosaceae	Woody	37.0 ± 11.9	46.7 ± 8.6

### Seed dispersal distances

The allometric models predicted long dispersal distances above a threshold of 1 km. Birds had a greater range of potential seed dispersal distances of 15.7 to 103.1 km, while the mammals’ dispersal distances ranged between 1.7 and 8.6 km.

## Discussion

The resilience of the critically endangered southern African grassland biome has been deteriorating, rendering it increasingly susceptible to the negative impacts of the non-native plants. In support of our prediction, frugivorous vertebrates are the main drivers of fleshy-fruited plant invasion in the grassland biome, dispersing disproportionately high numbers of seeds, leading to high propagule pressure and thus possible successful reinvasion of the biome by the study species: *P. angustifolia* and *O. engelmannii*. However, their seed dispersal effectiveness varied significantly, partly negatively impacting plant recruitment and invasion success. We show that knowledge of dispersal vector diversity and their dispersal effectiveness must be incorporated into the design of response management strategies. Beyond our results, we argue that, by considering the use of biological control methods, the number of dispersed seeds can be reduced and thus minimise the success of further invasions (Mokotjomela et al., 2015), particularly when seed dispersal distances by vertebrates are often substantial, enabling seeds to be deposited far from the maternal population targeted for control.

### Dispersal vectors and species diversity

The high vertebrate species diversity observed in the invaded areas compared with their non-invaded areas suggests a significant impact of vertebrates on the dispersal of invasive species, which could profoundly affect indigenous animal communities (Coetzee et al., 2007; Rieder et al., 2010; Vila et al., 2011; Stewart et al., 2021). Non-native species introduce novel resources, such as abundant and nutritious fruits, and provide refuge, resulting in ample food abundance to support a diverse array of species in the invaded grassland plant communities (Mokotjomela et al., 2013; Stewart et al., 2021). We suggest that the multiple dispersal vector species recorded in this study may provide various advantages that offset the negative impacts, such as seed predation (Vargas-Mendoza and González-Espinosa, 1992; Mokotjomela et al., 2016; Howe, 2016), diversification of dispersal routes, particularly the highly consumed plant species, and resilience against habitat loss (Howe, 2016; Sádlo et al., 2018) and specialised relationship loss (Traveset et al., 2014); Beckman & Sullivan, 2023). These advantages directly benefit plant species that rely heavily on animals for dispersal, because they provide alternative routes for seed dispersal. This vector diversity can enhance plant recruitment in various habitats, both for indigenous and non-native species (Jordano et al., 2007), because the overall effectiveness of seed dispersal depends on the combined interactions between different vector species and plant fruits and seeds (Dennis & Westcott, 2006; Nathan et al., 2008; Schurr et al., 2009; Mokotjomela et al., 2016). The more frequent visitation of *S. grimmia* and *B. taurus* on both study sites aligns with previous reports, which demonstrated that mammals in grassland ecosystems often display a preference for the encroaching non-native plant species and their associated rewards (Oosthuysen et al., 2023; Graham et al., 2020).

### Quantities of seeds dispersed

A substantial seed rain recorded in Boshof appeared to be associated with the presence of herbivorous/omnivorous mammals, whose diets contain fruits and seeds (Bunney et al., 2019); Howe, 1986; Jordano, 2000). Because mammals were dominant seed vectors in both study sites, they likely contributed to the disproportionate spread of the invasive non-native plants. Their foraging behaviour greatly enhances their opportunities of encountering, consuming, and dispersing the seeds of invasive plant species (Jordano, 2000; Higgins et al., 2003; Nathan et al., 2008; Tsoar et al., 2010); Stewart et al., 2021; Godó et al., 2022). In addition, foraging by avian species in both study areas can disperse seeds over long distances, introducing them into new microsites in the grassland (Gosper and Vivian‐Smith, 2009; Sethi and Howe, 2012; Mokotjomela et al., 2013). Mokotjomela et al. (2021) reported that birds of various sizes consume and effectively disperse two cacti species in an arid grassland habitat. In support, the spread of *P. angustifolia* in the grassland biome has been linked partly to foraging birds (Adams, 2020). It has been reported that *P. angustifolia* and *Opuntia engelmannii* have higher nutritional value (Adams et al., 2023; Sipango et al., 2022), which could have a significant influence on their consumption patterns. For example, *Pyracantha* fruits are generally rich in vitamin C and antioxidants (Zhang, 2020), and *Opuntia* fruits are a good source of fibre for animals in the arid region of the Americas (Dubeux et al., 2021), South Africa (Grobler et al., 2010; Aruwa et al., 2018; Sipango et al., 2022), Australia (Gouw, 2020), and parts of Africa and Asia (Elshehy et al., 2020). Fruit and seed characteristics are important determinants of vertebrate-mediated seed dispersal (Jordano, 2000; Schurr et al., 2009; Schupp et al., 2010). Vertebrates prefer fruits with medium-sized seeds, usually found in many non-native species, probably because they are easier to process than larger seeds, leading to quicker consumption and greater energy intake (Divíšek et al., 2018; Rejmanek & Richardson, 1996). Our findings of many small to medium-sized seeds in both study sites highlight high propagule pressure and possible further invasion (Lockwood et al., 2005). Similar to our results, Msweli (2021) found that mammal species contribute significantly to the seed dispersal of alien invasive plants, and that many birds heavily consumed invasive species, including common lantana *Lantana camara* L. (Verbenaceae) fruit in the Sub-Escarpment Grassland biome of KwaZulu-Natal, South Africa (Bitani & Downs, 2022). Many successful plant invaders globally have small to medium-sized seeds (Rejmanek & Richardson, 1996; Sakai et al., 2001; Schurr et al., 2009; Gosper and Vivian‐Smith, 2009), although small seeds are vulnerable to damage from predation during ingestion by vertebrates (Mokotjomela et al., 2016; McGowan, 2019; Carlin et al., 2024), because gustation can affect embryonic development (Meyer & Witmer, 1998; Miller et al., 2022; Traveset et al., 2008). Small seeds are also susceptible to extreme environmental conditions after being dispersed, diminishing efforts to colonise and establish long-lasting populations in new areas (Lesoli et al., 2013), and retarding invasion. Alternatively, a limited number of seeds gathered from uninvaded areas is likely due to the grassland habitats typically supporting few fruit-bearing plant species (Knight & Siegfried, 1983; Mucina and Rutherford, 2006; Canavan et al., 2022). Despite this, we observed that some seeds collected were still those of non-native species, suggesting a possible future invasion threat within the broader grasslands biome. Furthermore, the inherent fire regimes of the grassland biomes may suppress the recruitment of fleshy-fruited species (Calitz et al., 2015). Therefore, the apparent low invasion of these sites could be an artefact of regular grassland fires together with anthropogenic rangeland management (Vukeya et al., 2023). However, it is important to proactively monitor less invaded areas to prevent intensification of the invasion.

### Viability of dispersed seeds

The fruit pulp can hinder biochemical pathways of germination owing to the presence of compounds in the pulp that impede the successful initiation of seed germination (Meter and Witmer, 1998; Traveset et al., 2007; Chenyin et al., 2023). In support, Mokotjomela et al. (2021) found that the intact fruit of wheel cactus *Opuntia robusta* H.L. Wendl. ex Pfeiff. (Cactaceae) and prickly pear *Opuntia ficus-indica* (L.) Mill. (Cactaceae) demonstrated notably lower germination rates compared to treatments of de-pulped seeds, as well as seeds consumed by small and medium-sized birds in South Africa. De-pulped *O. engelmannii* seeds showed slightly enhanced germination rates compared with ingested seeds in our study, suggesting that this could be primarily centred on fruit pulp removal and seed coat modification, which the gut of vertebrates imposes physiological changes on the seeds that hinder germination (Herrera, 2002; Traveset & Verdú, 2002; Traveset et al., 2007). Similar results have been documented in *O. ficus-indica* seeds that passed through the guts of small birds (Mokotjomela et al., 2021). Padrón et al. (2011) also observed analogous findings with prickly pear cactus *Opuntia maxima* (Cactaceae) seeds processed through the gut of the yellow-legged gull *Larus michahellis* Naumann, JF, 1840 (Laridae). These findings suggest that the digestive processes can negatively impact seed germination, hindering plant population establishment.

Seed passage through a vertebrate’s gut is often linked to increased germination rates and seed dispersal in invasive plants (Traveset, 1998; Gosper et al., 2005; Corlett, 2017). For instance, the germination of *O. robusta* seeds was improved after being processed in the gut of avian frugivores in the southwestern grassland in Free State province, South Africa (Mokotjomela et al., 2021). In contrast, the *O. engelmannii* seeds displayed significantly lower viability (~ 5.4%) after ingestion, highlighting that the animals in our study did not improve seed germination of the invasive species, likely leading to reduced invasion potential, as also observed in sour prickly pear *Opuntia stricta* (Haw.) Haw. (Cactaceae) in Spain (Padrón et al., 2011). The ingestion may have caused abrasions that harm the embryo, thus low germination of *O. engelmannii* (Materechera & Materechera, 2001; Meyer & Witmer, 1998), and this also suggests that the seeds were sensitive to physical and chemical exposure, as reported for other cactus species (Rojas-Aréchiga and Vázquez-Yanes, 2000). Similarly, damage inflicted by seed consumers results in limited seed germination (Soler et al., 1993; Traveset et al., 2001 & 2008; Nogales et al., 2005; Mokotjomela et al., 2021). Vargas-Mendoza and González-Espinosa (1992) similarly observed that seeds of Sweet Prickly Pear *Opuntia streptacantha* Lem. (Cactaceae) ingested by rodents damages the seed embryo, potentially thwarting the species’ overall establishment. Nevertheless, this observation implies that, since many cacti species are vigorously invasive in South Africa (Mokotjomela et al., 2024b; Novoa et al., 2016; Walters et al., 2011&c), vegetative reproduction may contribute substantially to plant recruitment and population expansion, thereby leading to invasion.

The finding that *P. angustifolia* seeds retain relatively high viability even after ingestion by vertebrates could enhance their invasion potential in the grassland biomes. This observation aligns with the substantial germination rates exceeding 80% for *P. angustifolia* seeds consumed by various bird species (Adams et al., 2022). However, we suggest that the absence of significant differences in germination rates between de-pulped and ingested seeds indicates that passage through the vertebrate gut does not enhance germination of P. angustifolia. This outcome is consistent with findings by Adams et al. (2022), who reported similar results on farms in the eastern Free State province. This may be attributed to its hard seed coat (Darji et al., 2022), which may not have been effectively scarified during gut passage (Adams et al., 2022). Species with hard seed coats, such as tree of heaven *Ailanthus altissima* (Mill.) Swingle*.* (Simaroubaceae), silver wattle *A. dealbata* Link. (Fabaceae), and black locust *Robinia pseudoacacia* L. (Fabaceae), apparently contribute to their invasiveness (Cruz et al., 2021). Ungerminated *P. angustifolia* seeds do not maintain their dormancy and viability, with the highest survival rates recorded in the initial three months after burial, ranging from 90 to 98% (Adams et al., 2023). We suggest that *P. angustifolia* could benefit from de-pulping during the fruit consumption process, because the pulp impeded germination in certain plant species due to its sugar content, which can lead to a decrease in osmotic pressure and even prevent seed germination (Amodeo et al., 2017; Cipollini & Levey, 1997; Evenari, 1949; Traveset et al., 2007). Despite this, frugivores served a vital displacement function for seeds, effectively transporting seeds away from parent plants into novel environments, thus alleviating potentially exclusive inter- and intra-species competition (Chama et al., 2013; Mokotjomela et al., 2016; Vukeya et al., 2021).

### Seed dispersal distances

Long dispersal distances of the ingested non-native seeds confer recruitment advantages of low competition for the resources and thus development of new populations (Milton et al., 2007; Richardson et al., 2000; Sakai et al., 2001). Our model predictions also suggest that the observed dispersal vectors disperse seeds over long distances, possibly assisting the formation of new non-native populations in the southern African grassland biome. While the small mammals and residential birds usually traverse short distances due to their natural home ranges (McGregor et al., 2008; Nepali et al., 2024; Richard et al., 2022), other studies argue that these species can cover greater distances for specific needs, such as birds tracking fruit resources in scarce areas (Telleria et al., 2008) and escaping life-threatening events, i.e. fire, and predator risk and territory battle (Gomes et al., 2008; Mokotjomela et al., 2013). Indeed, the previous studies demonstrated that mammals and birds play a significant role in driving invasion within the southern African grassland biome (Adams et al., 2023; Canavan et al., 2022; Mokotjomela et al., 2021) although they did not determine the potential distance for seed dispersal which was reported as a critical factor in species movement across the geographical barriers and establishment (Richardson et al., 2000). Our study provides crucial insights into how to combat vertebrate-mediated plant invasions and thus enhance the ability to predict the establishment of new non-native populations and inform their management interventions.

### Management and policy implications

The conservation of the southern African grassland biome is subject to the national regulations enacted for non-native species in South Africa, which acknowledge their negative impacts on the environment, society, and economy, thereby warranting effective management. Consequently, the observed relatively high seed dispersal effectiveness in the study sites suggests that further invasions are possible and thus, their negative impacts. Although the risk assessment framework for listing non-native species in the national regulations in South Africa (i.e. Kumschick et al., 2020) considers whether species may invade the local area in the future, the framework does not have corresponding management recommendations for the containment of species’ movements. With emphasis on curbing the spread of the non-native species for containment of their negative impacts (van Wilgen et al., 2012; SANBI, 2017; IPBES, 2023; Sebeens et al., 2020), we argue that the local response strategies (i.e. management plans) for clearing non-native plant species populations within the grassland biome should be revised to integrate the movement ecology of the more preferred fruit-bearing species for early prevention of density-based effects from newly formed populations (Mokotjomela et al., 2024b; Pachepsky & Levine, 2011; Zhu et al., 2023). The successful germination and establishment of non-native plants can undermine the clearing efforts and cause further loss of the limited resources required for implementing management actions (Canavan et al., 2021; Mokotjomela et al., 2022). Cognisant that some propagule reservoirs can be located in inaccessible areas (Canavan et al., 2021; Mokotjomela et al., 2024a), management programmes must also consider concurrent use of biological control agents to reinforce mechanical methods (Pluess et al., 20,212), as recommended for the chemically resistant Pine-cone cactus *Tephrocactus articulatus* (Pfeiff.) (Cactaceae) in South Africa (Mokotjomela et al., 2024c). Biocontrol agents can also be easily integrated into other methods and are cheap to release systematically using records of the non-native target species population (Moran et al., 2013). Consistently, the number of seeds dispersed from primary populations was effectively reduced through the release of the seed-feeding weevil *Melanterius servulus* (Curculionidae) and the flower-galling midge *Dasineura dielsi* (Cecidomyiidae) on *Acacia cyclops* (Rooikrans, Fabaceae) in South Africa (Mokotjomela & Hoffmann, 2013). However, the release of biological control agents may require special consideration when the target non-native species holds conflicting societal or economic value (sensu Zengeya et al., 2017).

## Conclusions

This study demonstrated that the high plant invasion in the grassland biome is driven by the high diversity of vertebrate frugivores observed in invaded areas. In support, we recorded a substantial seed rain of non-native species, characterised by medium-sized seeds with hard coats. We contend that the widespread dispersal of *P. angustifolia* and *Opuntia engelmannii*, along with their potential introduction into novel environments, is particularly concerning because of inadequate current management efforts in the grassland biome. While the ingested seeds of *O. engelmannii* exhibited low viability, this does not negate the broader role of frugivore vertebrates in facilitating plant invasions. Rather, it underscores species-specific variation in seed response to gut passage, which may act as a natural filter on invasion success. The low germination of *O. engelmannii* suggests that not all non-native plant species benefit equally from frugivore vertebrate-mediated dispersal, highlighting the need to assess both the quantity and quality of dispersal events. This case may be viewed as an exception that supports the broader pattern that frugivore vertebrate-mediated dispersal contributes to invasion risk, but only where post-dispersal viability aligns with encounter rates and dispersal frequency. Our findings support the view that frugivore vertebrate-mediated dispersal contributes to the persistence and expansion of invasive plants, particularly in ecosystems with few indigenous fleshy-fruited species. Management strategies should therefore address both plants and animals of the invasion pathway, targeting priority non-native species while monitoring key frugivore vertebrate dispersers and their foraging behaviour (Buckley et al., 2006). Such integrated approaches are consistent with the 2030 goals of the Kunming–Montreal Global Biodiversity Framework. Restoration efforts should aim to reduce the availability or attractiveness of non-native fruits, promote indigenous plant cover, and enable early intervention in areas at risk of propagule accumulation. Finally, although our results suggest a strong link between frugivore vertebrate dispersal and invasion risk, they are correlative rather than experimental and thus should serve as a foundation for future mechanistic studies.

## Supplementary Information

Below is the link to the electronic supplementary material.

Supplementary file1 (DOCX 81.1 KB)

## Data Availability

No datasets were generated or analysed during the current study.

## Declarations

All authors have read, understood, and complied as applicable with the statement on “Ethical responsibilities of Authors” as found in the Instructions for Authors.
