# Supplementary material for: Seed dispersal by vertebrates promotes invasion risk in the southern African grassland biome
Source: Environ Monit Assess. 2025 Sep 19;197(10):1125. doi: 10.1007/s10661-025-14569-3 (PMC12449334; doi:10.1007/s10661-025-14569-3)
Supplement: Supplementary file 1 — Supplementary file1 (DOCX 81.1 KB) [file 10661_2025_14569_MOESM1_ESM.docx]

Supplementary information

Table S1: Species diversity documented in the southern African grassland in the invaded and non-invaded areas of Boshof and Fouriesburg towns from highest to lowest counts.

| **Species name** | **Common name** | **Feeding mode** | **Foraging frequency** | **% of the total** |
| --- | --- | --- | --- | --- |
| *Sylvicapra grimmia* | Common duiker | Herbivore | 69 | 21.04 |
| *Bos taurus* | Domestic cattle | Herbivore | 40 | 12.19 |
| *Ovis aries* | Domestic sheep | Herbivore | 31 | 9.45 |
| *Numida meleagris* | Helmeted guineafowl | Omnivore | 26 | 7.93 |
| *Dessonornis caffer* | Cape robin-chat | Insectivore | 17 | 5.18 |
| *Cynictis penicillata* | Yellow mongoose | Carnivore | 12 | 3.66 |
| *Lupulella mesomelas* | Black-backed jackal | Carnivore | 12 | 3.66 |
| *Herpestes sanguineus* | Common slender mongoose | Carnivore | 11 | 3.35 |
| *Lepus saxatilis* | Scrub hare | Herbivore | 9 | 2.74 |
| *Turdus olivaceus* | Olive Thrush | Frugivore | 9 | 2.74 |
| *Capra hircus* | Domestic goat | Herbivore | 8 | 2.44 |
| *Corvus albus* | Pied crow | Omnivore | 8 | 2.44 |
| *Streptopelia semitorquata* | Red-eyed dove | Granivore | 7 | 2.13 |
| *Aethomys namaquensis* | Namaqua rock mouse | Omnivore | 6 | 1.83 |
| *Proteles cristatus* | Aardwolf | Omnivore | 6 | 1.83 |
| *Hystrix africaeaustralis* | Cape porcupine | Omnivore | 4 | 1.22 |
| *Lanius collaris* | Southern fiscal | Omnivore | 4 | 1.22 |
| *Pycnonotus nigricans* | African red-eyed bulbul | Frugivore | 4 | 1.22 |
| *Raphicerus campestris* | Steenbok | Herbivore | 4 | 1.22 |
| *Bostrychia hagedash* | Hadeda Ibis | Omnivore | 3 | 0.95 |
| *Bubulcus ibis* | Cattle egret | Insectivore | 3 | 0.92 |
| *Colius striatus* | Speckled mousebird | Frugivore | 3 | 0.91 |
| *Columba guinea* | Speckled pigeon | Granivore | 3 | 0.91 |
| *Onychognathus morio* | Red-winged starling | Omnivore | 3 | 0.91 |
| *Pternistis afer* | Red-necked spurfowl | Insectivore | 3 | 0.91 |
| *Redunca redunca* | Bohor reedbuck | Graminivore | 2 | 0.61 |
| *Canis Lupus* | Australian native dog | Carnivore | 2 | 0.61 |
| *Ploceus velatus* | Southern masked weaver | Frugivore-Granivore | 2 | 0.61 |
| *Spilopelia senegalensis* | Laughing Dove | Granivore | 2 | 0.61 |
| *Stigmochelys pardalis* | Leopard tortoise | Herbivore | 2 | 0.61 |
| *Chlorocebus pygerythrus* | Vervet monkey | Omnivore | 1 | 0.31 |
| *Civettictis civetta* | African civet | Omnivore | 1 | 0.31 |
| *Colius colius* | White-backed mousebird | Frugivore | 1 | 0.31 |
| *Dicrurus adsimilis* | Fork-tailed drongo | Frugivore | 1 | 0.31 |
| *Lamprotornis bicolor* | Pied starling | Omnivore | 1 | 0.31 |
| *Mellivora capensis* | Honey badger | Carnivore | 1 | 0.31 |
| *Papio ursinus* | Chacma baboon | Omnivore | 1 | 0.31 |
| *Phoeniculus purpureus* | Green wood-hoopoe | Omnivore | 1 | 0.31 |
| *Columba guinea* | Speckled Pigeon | Granivores | 1 | 0.31 |
| *Pycnonotus tricolor* | Dark-capped bulbul | Frugivore | 1 | 0.31 |
| *Streptopelia capicola* | Ring-necked dove | Granivore | 1 | 0.31 |
| *Sturnus unicolor* | Spotless starling | Omnivore | 1 | 0.31 |
| *Thryonomys swinderianus* | Greater cane rat | Herbivore | 1 | 0.31 |

Table S2. Non-native species diversity recorded in the grassland biome. The data was gathered from several reputable platforms, including Global Biodiversity Information Facility (GBIF), iNaturalist, and the Southern African Plant Invader Atlas (SAPIA), ensuring a comprehensive and reliable set of records and were cleaned using cleaned using the package Biogeo in R version 4.1.0.

| Species | Family | Plant life form | |
| --- | --- | --- | --- |
| *Abelia* × *grandiflora* (Andre) Rehder | Caprifoliaceae | | Woody |
| *Abelia chinensis* R.Br. | Caprifoliaceae | | Woody |
| *Abies religiosa* (Kunth) Cham. & Schltdl. | Pinaceae | | Woody |
| *Abutilon* × *hybridum* Siebert & Voss | Malvaceae | | Woody |
| *Abutilon grandifolium* (Willd.) Sweet | Malvaceae | | Woody |
| *Abutilon theophrasti* Medik. | Malvaceae | | Herb |
| *Acacia baileyana* F. Muell. | Fabaceae | | Woody |
| *Acacia cultriformis* A.Cunn. ex G.Don | FabaceaeFabaceaeFabaceaeFabace Fabaceae | | Woody |
| *Acacia cyclops* A.Cunn. ex G.Don | Fabaceae | | Woody |
| *Acacia dealbata* Link | Fabaceae | | Woody |
| *Acacia decurrens* Willd. | Fabaceae | | Woody |
| *Acacia elata* A.Cunn. ex Benth. | Fabaceae | | Woody |
| *Acacia longifolia* (Andrews) Willd. | Fabaceae | | Woody |
| *Acacia mearnsii* De Wild. | Fabaceae | | Woody |
| *Acacia melanoxylon* R.Br. | Fabaceae | | Woody |
| *Acacia paradoxa* DC. | Fabaceae | | Woody |
| *Acacia pendula* A.Cunn. ex G.Don | Fabaceae | | Woody |
| *Acacia podalyriifolia* A.Cunn. ex G.Don | Fabaceae | | Woody |
| Acacia pycnantha Benth. | Fabaceae | | Woody |
| Acacia saligna (Labill.) H.L.Wendl. | Fabaceae | | Woody |
| *Acalypha indica* var. bailloniana (Mull.Arg.) Hutch. | Euphorbiaceae | | Herb |
| *Acalypha punctata* var. longifolia Prain | Euphorbiaceae | | Herb |
| *Acalypha punctata* var. rogersii Prain | Euphorbiaceae | | Herb |
| *Acanthospermum australe* (Loefl.) Kuntze | Asteraceae | | Herb |
| *Acanthospermum glabratum* (DC.) Wild | Asteraceae | | Herb |
| *Acanthospermum hispidum* DC. | Asteraceae | | Herb |
| *Acanthus mollis* L. | Acanthaceae | | Herb |
| *Acanthus polystachyus* Delile | Acanthaceae | | Woody |
| *Acanthus pubescens* (Thomson ex Oliv.) Engl. | Acanthaceae | | Woody |
| *Acer buergerianum* Miq. | Aceraceae | | Woody |
| *Acer griseum* (Franch.) Pax | Aceraceae | | Woody |
| *Acer negundo* L. | Aceraceae | | Woody |
| *Acer pseudoplatanus* L. 'Atropurpureum' | Aceraceae | | Woody |
| *Achillea millefolium* L. | Asteraceae | | Herb |
| *Achyranthes aspera* L. var. aspera | Amaranthaceae | | Herb |
| *Acmella caulirhiza* Delile | Asteraceae | | Herb |
| *Acmella decumbens* (Sm.) R.K.Jansen | Asteraceae | | Herb |
| Acorus calamus L. | Acoraceae | | Herb |
| Acrocarpus fraxinifolius Wight & Arn. | Fabaceae | | Woody |
| *Acrotome fleckii* (Gurke) Launert | Lamiaceae | | Herb |
| *Actinidia deliciosa* (A.Chev.) C.F.Liang & A.R.Ferguson | Actinidiaceae | | Woody |
| *Adenostemma viscosum* J.R.Forst. & G.Forst. | Asteraceae | | Herb |
| *Adiantum hispidulum* var. hispidulum | Pteridaceae | | Herb |
| *Adiantum raddianum* C.Presl | Pteridaceae | | Herb |
| *Adonis aestivalis* L. | Ranunculaceae | | Herb |
| *Aesculus hippocastanum* L. | Sapindaceae | | Woody |
| *Agathis dammara* (Lamb.) Rich. | Araucariaceae | | Woody |
| *Agathis robusta* (F.Muell.) F.M.Bailey | Araucariaceae | | Woody |
| *Agathisanthemum bojeri* subsp. angolense (Bremek.) Verdc. | Rubiaceae | | Woody |
| *Agave americana* L. subsp. americana var. americana | Asparagaceae | | Succulent |
| *Agave americana* L. var. expansa (Jacobi) Gentry | Asparagaceae | | Succulent |
| *Agave angustifolia* var. angustifolia | Asparagaceae | | Succulent |
| *Agave sisalana* Perrine | Asparagaceae | | Succulent |
| *Agave wercklei* F.A.C.Weber ex Werckle | Asparagaceae | | Succulent |
| *Ageratina adenophora* (Spreng.) R.M. King & H. Robinson | Asteraceae | | Herb |
| *Ageratina riparia* (Regel) R.King & H.Robinson | Asteraceae | | Herb |
| *Ageratum conyzoides* (L.) L. | Asteraceae | | Herb |
| *Ageratum houstonianum* Mill. | Asteraceae | | Herb |
| *Agrimonia procera* Wallr. | Rosaceae | | Herb |
| *Agrostis gigantea* Roth | Poaceae | | Grass |
| *Agrostis montevidensis* Spreng. ex Nees | Poaceae | | Grass |
| *Ailanthus altissima* (Mill.) Swingle | Simaroubaceae | | Woody |
| *Aira cupaniana* Guss. | Poaceae | | Grass |
| *Albizia chinensis* (Osbeck) Merr. | Fabaceae | | Woody |
| *Albizia julibrissin* Durazz. | Fabaceae | | Woody |
| *Albizia lebbeck* (L.) Benth. | Fabaceae | | Woody |
| *Albizia procera* (Roxb.) Benth. | Fabaceae | | Woody |
| *Alectra sessiliflora* var. monticola (Engl.) Melch. | Scrophulariaceae | | Herb |
| *Alectra thyrsoidea* Melch. | Scrophulariaceae | | Herb |
| *Aleurites moluccanus* (L.) Willd. | Euphorbiaceae | | Woody |
| *Aleurites moluccanus* var. moluccanus | Euphorbiaceae | | Woody |
| *Alisma plantago-aquatica* L. | Alismataceae | | Herb |
| *Allamanda cathartica* L. | Apocynaceae | | Woody |
| *Allocasuarina torulosa* (Aiton) L.A.S.Johnson | Casuarinaceae | | Woody |
| *Alnus glutinosa* (L.) Gaertn. | Betulaceae | | Woody |
| *Alnus rubra* Bong. | Betulaceae | | Woody |
| *Alocasia macrorrhizos* (L.) G. Don | Araceae | | Herb |
| *Aloe polyphylla* Schonland ex Pillans | Asphodelaceae | | Succulent |
| *Aloe welwitschii* Klopper & Gideon F.Sm. | Asphodelaceae | | Succulent |
| *Alopecurus arundinaceus* Poir. | Poaceae | | Succulent |
| *Alpinia zerumbet* (Pers.) B.L. Burtt & R.M. Sm. | Zingiberaceae | | Herb |
| *Alstroemeria pulchella* L.f. | Alstroemeriaceae | | Woody |
| *Alternanthera caracasana* Kunth | Amaranthaceae | | Herb |
| *Alternanthera pungens* Kunth | Amaranthaceae | | Herb |
| *Alternanthera sessilis* (L.) R.Br. ex DC. | Amaranthaceae | | Herb |
| *Amaranthus deflexus* L. | Amaranthaceae | | Herb |
| *Amaranthus dubius* Mart. ex Thell. | Amaranthaceae | | Herb |
| *Amaranthus graecizans* L. subsp. graecizans | Amaranthaceae | | Herb |
| *Amaranthus hybridus* L. | Amaranthaceae | | Herb |
| *Amaranthus hybridus* L. subsp. cruentus (L.) Thell. | Amaranthaceae | | Herb |
| *Amaranthus hybridus* L. subsp. hybridus var. hybridus | Amaranthaceae | | Herb |
| *Amaranthus hybridus* subsp. hybridus var. erythrostachys Moq. | Amaranthaceae | | Herb |
| *Amaranthus lividus* subsp. polygonoides (Moq.) Probst | Amaranthaceae | | Herb |
| *Amaranthus spinosus* L. | Amaranthaceae | | Herb |
| *Amaranthus viridis* L. | Amaranthaceae | | Herb |
| *Ambrosia artemisiifolia* var. artemisiifolia | Asteraceae | | Herb |
| *Ambrosia psilostachya* DC. | Asteraceae | | Herb |
| *Amelichloa clandestina* (Hack.) Arriaga & Barkworth | Poaceae | | Grass |
| *Ammi majus* L. | Apiaceae | | Woody |
| *Amorphophallus campanulatus* (Roxb.) Blume | Araceae | | Herb |
| *Ampelocissus africana* (Lour.) Merr. var. africana | Vitaceae | | Herb |
| *Amsinckia menziesii* (Lehm.) A.Nelson & J.F.Macbr. | Boraginaceae | | Herb |
| *Ananas comosus* (L.) Merr. | Bromeliaceae | | Herb |
| *Anchusa azurea* Mill. | Boraginaceae | | Herb |
| *Andropogon brazzae* Franch. | Poaceae | | Grass |
| *Aneura pinguis* (L.) Dumort. | Aneuraceae | | Herb |
| *Angophora costata* (Gaertn.) Britten | Myrtaceae | | Woody |
| *Anoda cristata* (L.) Schltdl. | Malvaceae | | Woody |
| *Anredera baselloides* (Kunth) Baill. [1 | Basellaceae | | Herb |
| *Anredera cordifolia* (Ten.) Steenis | Basellaceae | | Herb |
| *Anthemis arvensis* L. | Asteraceae | | Herb |
| *Anthemis cotula* L. | Asteraceae | | Herb |
| *Anthriscus sylvestris* (L.) Hoffm. var. sylvestris | Apiaceae | | Herb |
| *Antidesma rufescens* Tul. | Euphorbiaceae | | Woody |
| *Antigonon leptopus* Hook. & Arn. | Polygonaceae | | Herb |
| *Apium graveolens* L. | Apiaceae | | Herb |
| *Apium prostratum* Vent. | Apiaceae | | Herb |
| *Arachis hypogaea* L. | Fabaceae | | Herb |
| *Aralia spinosa* L. | Araliaceae | | Woody |
| *Araucaria bidwillii* Hook. | Araucariaceae | | Woody |
| *Araucaria heterophylla* (Salisb.) Franco | Araucariaceae | | Woody |
| *Araucaria rulei* F.Muell. | Araucariaceae | | Woody |
| *Araujia sericifera* Brot. | Apocynaceae | | Herb |
| *Archontophoenix cunninghamiana* (H.Wendl.) H.Wendl. & Drude | Arecaceae | | Woody |
| *Ardisia crenata* Sims | Myrsinaceae | | Woody |
| *Arenaria serpyllifolia* L. | Caryophyllaceae | | Herb |
| *Argemone mexicana* L. | Papaveraceae | | Herb |
| *Argemone ochroleuca* Sweet subsp. ochroleuca | Papaveraceae | | Herb |
| *Argyreia nervosa* (Burm.f.) Bojer | Convolvulaceae | | Herb |
| *Aristida hordeacea* Kunth | Poaceae | | Grass |
| *Aristida scabrivalvis* subsp. borumensis (Henrard) Melderis | Poaceae | | Grass |
| *Aristolochia labiata* Willd. | Aristolochiaceae | | Herb |
| *Aristolochia littoralis* Parodi | Aristolochiaceae | | Herb |
| *Aristolochia macrophylla* Lam. | Aristolochiaceae | | Herb |
| *Aristolochia ridicula* N.E.Br. | Aristolochiaceae | | Herb |
| *Arrhenatherum elatius* (L.) P.Beauv. ex J.Presl & C.Presl | Poaceae | | Grass |
| *Artemisia vulgaris* L. | Asteraceae | | Herb |
| Arthropteris orientalis (J.F.Gmel.) Posth. | Oleandraceae | | Fern |
| Arum palaestinum Boiss. | Araceae | | Bulb |
| Arundinella nepalensis Trin. | Poaceae | | Grass |
| *Arundo donax* L. | Poaceae | | Grass |
| *Asclepias curassavica* L. | Apocynaceae | | Herb |
| *Aspidoglossum lanatum* (Weim.) Kupicha | Apocynaceae | | Herb |
| *Asplenium trichomanes* L.subsp. trichomanes | Aspleniaceae | | Herb |
| *Asplenium trichomanes* subsp. quadrivalens D.E.Mey. | Aspleniaceae | | Fern |
| *Aster novi-belgii* L. | Asteraceae | | Herb |
| *Astragalus bisulcatus* (Hook.) A.Gray | Fabaceae | | Herb |
| *Asystasia gangetica* (L.) T.Anderson subsp. gangetica | Acanthaceae | | Herb |
| *Athrixia rosmarinifolia* (Sch.Bip.) Oliv. & Hiern var. rosmarinifolia | Chenopodiaceae | | Herb |
| *Atriplex inflata* F.Muell. | Chenopodiaceae | | Herb |
| *Atriplex lindleyi* subsp. inflata (F.Muell.) Paul G.Wilson | Chenopodiaceae | | Herb |
| *Atriplex muelleri* Benth. | Chenopodiaceae | | Herb |
| *Atriplex nummularia* Lindl. subsp. nummularia | Chenopodiaceae | | Woody |
| *Atriplex patula* subsp. austro-africana Aellen | Chenopodiaceae | | Herb |
| *Atriplex semibaccata* R.Br. | Chenopodiaceae | | Herb |
| *Atriplex spongiosa* F.Muell. | Chenopodiaceae | | Herb |
| *Atriplex suberecta* I.Verd. | Chenopodiaceae | | Herb |
| *Austrocylindropuntia cylindrica* (Juss. ex Lam.) Backeb. | Cactaceae | | Succulent |
| *Austrocylindropuntia subulata* (Muehlenpf.) Backeb. | Cactaceae | | Succulent |
| *Avena byzantina* K.Koch | Poaceae | | Grass |
| *Avena fatua* L. | Poaceae | | Grass |
| *Avena sativa* L. | Poaceae | | Grass |
| *Axonopus fissifolius* (Raddi) Kuhlm. | Poaceae | | Herb |
| *Azolla cristata* Kaulf. | Azollaceae | | Fern |
| *Azolla filiculoides* Lam. | Azollaceae | | Fern |
| *Azolla microphylla* Kaulf. | Azollaceae | | Fern |
| *Azolla pinnata* R.Br. subsp. asiatica R.M.K.Saunders & K.Fowler | Azollaceae | | Fern |
| *Azolla pinnata* subsp. africana (Desv.) R.M.K.Saunders & K.Fowler | Azollaceae | | Fern |
| *Baccharis pingraea* DC. | Asteraceae | | Woody |
| *Baccharoides adoensis* (Sch.Bip. ex Walp.) H.Rob. | Asteraceae | | Woody |
| *Baillonella toxisperma* var. obovata Aubrev. & Pellegr. | Sapotaceae | | Woody |
| *Bambusa balcooa* Roxb. ex Roxb. | Poaceae | | Grass |
| *Bambusa vulgaris* Schrad. ex J.C.Wendl. | Poaceae | | Grass |
| *Banksia ericifolia* L.f. | Proteaceae | | Woody |
| *Banksia grandis* Willd. | Proteaceae | | Woody |
| *Banksia integrifolia* subsp. integrifolia | Proteaceae | | Woody |
| *Banksia serrata* L.fil. | Proteaceae | | Woody |
| *Banksia spinulosa* var. collina (R.Br.) A.S.George | Proteaceae | | Woody |
| *Barbarea verna* (Mill.) Asch. | Brassicaceae | | Herb |
| *Bartlettina sordida* (Less.) R.M.King & H.Rob. | Asteraceae | | Woody |
| *Bartsia trixago* L. | Orobanchaceae | | Herb |
| *Bassia indica* (Wight) A.J.Scott | Amaranthaceae | | Herb |
| *Bauhinia forficata* Link | Fabaceae | | Woody |
| *Bauhinia petersiana* Bolle subsp. petersiana | Fabaceae | | Woody |
| *Bauhinia petersiana* subsp. macrantha (Oliv.) Brummitt & J.H.Ross | Fabaceae | | Woody |
| *Bauhinia purpurea* L. | Fabaceae | | Woody |
| *Bauhinia variegata* L. 'Candida' | Fabaceae | | Woody |
| *Bauhinia variegata* var. variegata | Fabaceae | | Woody |
| *Begonia acutifolia* Dryand. | Begoniaceae | | Woody |
| *Begonia cucullata* Willd. | Begoniaceae | | Herb |
| *Begonia salisburyana* Irmsch. | Begoniaceae | | Woody |
| *Berberis* × *stenophylla* Lindl. | Berberidaceae | | Woody |
| *Berberis julianae* C.K.Schneid. | Berberidaceae | | Woody |
| *Berberis thunbergii* DC. 'Atropurpurea' | Berberidaceae | | Woody |
| *Berberis vulgaris* L. | Berberidaceae | | Woody |
| *Berberis wilsoniae* Hemsl. | Berberidaceae | | Woody |
| *Beta vulgaris* subsp. vulgaris | Amaranthaceae | | Herb |
| *Betula nigra* L. | Betulaceae | | Woody |
| *Betula pendula* Roth | Betulaceae | | Woody |
| *Biancaea decapetala* (Roth) O.Deg. | Fabaceae | | Woody |
| *Bidens bipinnata* L. | Asteraceae | | Herb |
| *Bidens biternata* (Lour.) Merr. & Sherff | Asteraceae | | Herb |
| *Bidens pilosa* var. pilosa | Asteraceae | | Herb |
| *Bixa orellana* L. | Bixaceae | | Woody |
| *Blepharis* hybrid | Acanthaceae | | Woody |
| *Blitum virgatum* L. subsp. virgatum | Amaranthaceae | | Herb |
| *Bocconia frutescens* L. | Papaveraceae | | Woody |
| *Boerhavia cordobensis* Kuntze | Nyctaginaceae | | Herb |
| *Boerhavia diffusa* L. var. diffusa | Nyctaginaceae | | Herb |
| *Boerhavia erecta* L. | Nyctaginaceae | | Herb |
| *Borago officinalis* L. | Boraginaceae | | Herb |
| *Bougainvillea* × *buttiana* Holttum & Standl. | Nyctaginaceae | | Woody |
| *Bougainvillea glabra* Choisy | Nyctaginaceae | | Woody |
| *Bougainvillea peruviana* Humb. & Bonpl. | Nyctaginaceae | | Woody |
| *Bougainvillea spectabilis* Willd. | Nyctaginaceae | | Woody |
| *Brachiaria advena* Vickery | Poaceae | | Grass |
| *Brachychiton acerifolium* (A.Cunn.) F.Muell. | Malvaceae | | Woody |
| *Brachychiton discolor* F.Muell. | Malvaceae | | Woody |
| *Brachychiton populneus* (Schott & Endl.) R.Br. | Malvaceae | | Woody |
| *Brachychiton rupestris* (Lindl.) K.Schum. | Malvaceae | | Woody |
| *Brachypodium distachyon* (L.) P.Beauv. | Poaceae | | Grass |
| *Brassica elongata* Ehrh. subsp. elongata | Brassicaceae | | Herb |
| *Brassica juncea* (L.) Czern. | Brassicaceae | | Herb |
| *Brassica nigra* (L.) W.D.J.Koch | Brassicaceae | | Herb |
| *Brassica rapa* L. | Brassicaceae | | Herb |
| *Brassica tournefortii* Gouan | Brassicaceae | | Herb |
| *Breynia disticha* J.R.Forst. & G.Forst. | Euphorbiaceae | | Woody |
| *Brillantaisia kirungae* Lindau | Acanthaceae | | Woody |
| *Briza maxima* L. | Poaceae | | Grass |
| *Briza minor* L. | Poaceae | | Grass |
| *Briza subaristata* Lam. | Poaceae | | Grass |
| *Bromus catharticus* Vahl | Poaceae | | Grass |
| *Bromus commutatus* Schrad. | Poaceae | | Grass |
| *Bromus diandrus* Roth | Poaceae | | Grass |
| *Bromus firmior* (Nees) Stapf | Poaceae | | Grass |
| *Bromus hordeaceus* subsp. molliformis (J.Lloyd) Maire & Weiller | Poaceae | | Grass |
| *Bromus inermis* Leyss. | Poaceae | | Grass |
| *Bromus leptoclados* Nees | Poaceae | | Grass |
| *Bromus madritensis* L. | Poaceae | | Grass |
| *Bromus natalensis* Stapf | Poaceae | | Grass |
| *Bromus pectinatus* Thunb. | Poaceae | | Grass |
| *Bromus speciosus* Nees | Poaceae | | Grass |
| *Brugmansia arborea* (L.) Steud. | Solanaceae | | Woody |
| *Brugmansia arborea* (L.) Sweet | Solanaceae | | Woody |
| *Brugmansia aurea* (Lagerh.) Saff. | Solanaceae | | Woody |
| *Brugmansia suaveolens* (Willd.) Sweet | Solanaceae | | Woody |
| *Brunfelsia pauciflora* (Cham. & Schltdl.) Benth. 'Eximia' | Solanaceae | | Woody |
| *Bryophyllum delagoense* (Eckl. & Zeyh.) Druce | Crassulaceae | | Succulent |
| *Buddleja madagascariensis* Lam. | Scrophulariaceae | | Woody |
| *Buglossoides arvensis* (L.) I.M.Johnst. | Boraginaceae | | Herb |
| *Buglossoides arvensis* subsp. arvensis | Boraginaceae | | Herb |
| *Bupleurum rotundifolium* L. | Apiaceae | | Herb |
| *Butea monosperma* (Lam.) Taub. | Fabaceae | | Woody |
| *Caesalpinia decapetala* (Roth) Alston | Fabaceae | | Woody |
| *Caesalpinia gilliesii* (Hook.) D. Dietr. | Fabaceae | | Woody |
| *Caesalpinia pulcherrima* (L.) Sw. | Fabaceae | | Woody |
| *Cajanus cajan* (L.) Huth | Fabaceae | | Woody |
| *Cajanus cajan* (L.) Millsp. | Fabaceae | | Woody |
| *Calendula arvensis* L. | Asteraceae | | Herb |
| *Calliandra tweedii* Benth. | Fabaceae | | Woody |
| *Callisia fragrans* (Lindl.) Woodson | Commelinaceae | | Herb |
| *Callisia repens* (Jacq.) L. | Commelinaceae | | Herb |
| *Callistemon citrinus* (Curtis) Skeels | Myrtaceae | | Woody |
| *Callistemon viminalis* subsp. viminalis | Myrtaceae | | Woody |
| *Callitris columellaris* F.Muell. | Cupressaceae | | Woody |
| *Callitris rhomboidea* R.Br. ex Rich. | Cupressaceae | | Woody |
| *Callitris verrucosa* (A.Cunn. ex Endl.) F.Muell. | Cupressaceae | | Woody |
| *Calocedrus decurrens* (Torr.) Florin | Cupressaceae | | Woody |
| *Calotropis procera* (Aiton) W.T.Aiton | Apocynaceae | | Woody |
| *Camellia sinensis* (L.) Kuntze | Theaceae | | Woody |
| *Campuloclinium macrocephalum* (Less.) DC. | Asteraceae | | Herb |
| *Campyliadelphus chrysophyllus* (Brid.) R.S.Chopra | Amblystegiaceae | | Herb |
| *Canavalia gladiata* (Jacq.) DC. | Fabaceae | | Herb |
| *Canna* × *generalis* L.H.Bailey | Cannaceae | | Herb |
| *Canna flaccida* Salisb. | Cannaceae | | Herb |
| *Canna glauca* L. | Cannaceae | | Herb |
| *Canna indica* L. | Cannaceae | | Herb |
| *Cannabis sativa* L. var. sativa | Cannabaceae | | Herb |
| *Cannabis sativa* subsp. indica (Lam.) E.Small & Cronquist | Cannabaceae | | Herb |
| *Cannabis sativa* var. indica (Lam.) Wehmer | Cannabaceae | | Herb |
| *Cannabis sativa* var. ruderalis (Janisch.) S.Z.Liou | Cannabaceae | | Herb |
| *Cantinoa americana* (Aubl.) Harley & J.F.B.Pastore | Lamiaceae | | Woody |
| *Cantinoa mutabilis* (Rich.) Harley & J.F.B.Pastore | Lamiaceae | | Woody |
| *Capsella bursa-pastoris* (L.) Medik. | Brassicaceae | | Herb |
| *Capsicum annuum* L. | Solanaceae | | Woody |
| *Capsicum annuum* var. glabriusculum (Dunal) Heiser & Pickersgill | Solanaceae | | Herb |
| *Capsicum frutescens* L. | Solanaceae | | Herb |
| *Cardamine flexuosa* With. | Brassicaceae | | Herb |
| *Cardamine hirsuta* L. | Brassicaceae | | Herb |
| *Cardamine impatiens* L. | Brassicaceae | | Herb |
| *Cardiospermum grandiflorum* Sw. | Sapindaceae | | Herb |
| *Cardiospermum halicacabum* L. var. halicacabum | Sapindaceae | | Herb |
| *Cardiospermum halicacabum* var. microcarpum (Kunth) Blume | Sapindaceae | | Herb |
| *Carduus macrocephalus* Desf. | Asteraceae | | Herb |
| *Carduus nutans* L. | Asteraceae | | Herb |
| *Carduus tenuiflorus* Curtis | Asteraceae | | Herb |
| *Carex acutiformis* Ehrh. | Poaceae | | Grass |
| *Carex divisa* Huds. | Poaceae | | Grass |
| *Carex* hybrid | Poaceae | | Grass |
| *Carex sylvatica* Huds. | Poaceae | | Grass |
| *Carica papaya* L. | Caricaceae | | Woody |
| *Caryopteris odorata* (Ham.) Rob. | Lamiaceae | | Woody |
| *Cascabela thevetia* (L.) Lippold | Apocynaceae | | Woody |
| *Casimiroa edulis* La Llave | Rutaceae | | Woody |
| *Cassia fistula* L. | Fabaceae | | Woody |
| *Cassia italica* (Mill.) Lam. ex F.W.Andrews subsp. italica | Fabaceae | | Woody |
| *Castanea dentata* (Marsh) Borkh. | Fagaceae | | Woody |
| *Castanea sativa* Mill. | Fagaceae | | Woody |
| *Castanospermum australe* A.Cunn. & Fraser | Fabaceae | | Woody |
| *Casuarina cunninghamiana* Miq. | Casuarinaceae | | Woody |
| *Casuarina equisetifolia* L. | Casuarinaceae | | Woody |
| *Catharanthus roseus* (L.) G.Don | Apocynaceae | | Herb |
| *Catinaria laureri* (Hepp ex Th.Fr.) Degel. | Bacidiaceae | | Herb |
| *Caucalis platycarpos* L. | Apiaceae | | Herb |
| *Cecropia peltata* L. | Cecropiaceae | | Woody |
| *Cedrela odorata* L. | Meliaceae | | Woody |
| *Cedrus deodara* (D.Don) G.Don | Pinaceae | | Woody |
| *Ceiba speciosa* (A.St.-Hil., A.Juss. & Cambess.) Ravenna | Malvaceae | | Woody |
| *Celtis australis* L. | Celtidaceae | | Woody |
| *Celtis sinensis* Pers. | Celtidaceae | | Woody |
| *Cenchrus brownii* Roem. & Schult. | Poaceae | | Grass |
| *Cenchrus geniculatus* Thunb. | Poaceae | | Grass |
| *Cenchrus setaceus* (Forssk.) Morrone | Poaceae | | Grass |
| *Cenchrus spinifex* Cav. | Poaceae | | Herb |
| *Centaurea calcitrapa* L. | Asteraceae | | Herb |
| *Centaurea cyanus* L. | Asteraceae | | Herb |
| *Centaurea melitensis* L. | Asteraceae | | Herb |
| *Centaurea nigra* L. | Asteraceae | | Herb |
| *Centaurea solstitialis* L. | Asteraceae | | Herb |
| *Centranthus ruber* (L.) DC. | Caprifoliaceae | | Herb |
| *Centratherum punctatum* Cass. subsp. punctatum | Asteraceae | | Herb |
| *Cephalozia bicuspidata* (L.) Dumort. | Cephaloziaceae | | Herb |
| *Cerastium fontanum* subsp. vulgare (Hartm.) Greuter & Burdet | Caryophyllaceae | | Herb |
| *Cerastium glomeratum* Thuill. | Caryophyllaceae | | Herb |
| *Ceratonia siliqua* L. | Fabaceae | | Woody |
| *Ceratophyllum demersum* L. | Ceratophyllaceae | | Herb |
| *Ceratophyllum demersum* L. var. demersum | Ceratophyllaceae | | Herb |
| *Cereus hildmannianus* K.Schum. | Cactaceae | | Succulent |
| *Cereus jamacaru* DC. | Cactaceae | | Succulent |
| *Cestrum aurantiacum* Lindl. | Solanaceae | | Woody |
| *Cestrum elegans* (Brongn. ex Neumann) Schltdl. | Solanaceae | | Woody |
| *Cestrum laevigatum* Schltdl. | Solanaceae | | Woody |
| *Cestrum parqui* (Lam.) L'Herit. | Solanaceae | | Woody |
| *Chamaecyparis lawsoniana* (A.Murray) Parl. | Cupressaceae | | Woody |
| *Chamaecyparis obtusa* (Siebold & Zucc.) Endl. 'Filicoides' | Cupressaceae | | Woody |
| *Chamaecyparis pisifera* (Siebold & Zucc.) Endl. | Cupressaceae | | Woody |
| *Chamaelaucium uncinatum* Schauer | Myrtaceae | | Woody |
| *Chenopodiastrum murale* (L.) S.Fuentes, Uotila & Borsch | Amaranthaceae | | Herb |
| *Chenopodium* × *bontei* Aellen | Amaranthaceae | | Herb |
| *Chenopodium album* L. | Amaranthaceae | | Herb |
| *Chenopodium amboanum* (Murr) Aellen | Amaranthaceae | | Herb |
| *Chenopodium giganteum* D.Don | Amaranthaceae | | Herb |
| *Chenopodium hircinum* Schrad. | Amaranthaceae | | Herb |
| *Chenopodium opulifolium* Schrad. ex W.D.J.Koch & Ziz | Amaranthaceae | | Herb |
| *Chenopodium strictum* Roth | Amaranthaceae | | Herb |
| *Chionanthus virginicus* L. | Oleaceae | | Woody |
| *Chloris gayana* Kunth | Poaceae | | Grass |
| *Chloris pycnothrix* Trin. | Poaceae | | Grass |
| *Chloris virgata* Sw. | Poaceae | | Grass |
| *Chondrilla juncea* L. | Asteraceae | | Herb |
| *Chromolaena odorata* (L.) R.M. King & H. Robinson | Asteraceae | | Woody |
| *Chrysojasminum humile* (L.) Banfi | Oleaceae | | Woody |
| *Chrysopogon nigritanus* (Benth.) Veldkamp | Poaceae | | Grass |
| *Cibotium glaucum* (Sm.) Hook. & Arn. | Asteraceae | | Woody |
| *Cichorium intybus* L. subsp. intybus | Asteraceae | | Herb |
| *Cienfuegosia gerrardii* (Harv.) Hochr. | Malvaceae | | Herb |
| *Cineraria anampoza* (Baker) Baker f. anampoza | Compositae | | Herb |
| *Cinnamomum camphora* (L.) J. Presl. | Lauraceae | | Woody |
| *Cinnamomum verum* J.Presl | Lauraceae | | Woody |
| *Cirsium arvense* (L.) Scop. | Asteraceae | | Herb |
| *Cirsium vulgare* (Savi) Ten. | Asteraceae | | Herb |
| *Cirsium vulgare* subsp. vulgare | Asteraceae | | Herb |
| *Citharexylum spinosum* L. | Verbenaceae | | Woody |
| *Citrus limon* (L.) Burm.f. | Rutaceae | | Woody |
| *Citrus sinensis* (L.) Osbeck | Rutaceae | | Woody |
| *Clematis gouriana* Roxb. ex DC. | Ranunculaceae | | Woody |
| *Clerodendrum bungei* Steud. | Lamiaceae | | Woody |
| *Clerodendrum floribundum* R.Br. | Lamiaceae | | Woody |
| *Clerodendrum fragrans* var. pleniflorum Schauer | Lamiaceae | | Woody |
| *Clerodendrum quadriloculare* (Blanco) Merr. | Lamiaceae | | Woody |
| *Clerodendrum splendens* G.Don | Lamiaceae | | Woody |
| *Clerodendrum thomsoniae* Balf. | Lamiaceae | | Woody |
| *Clerodendrum ugandense* Prain | Lamiaceae | | Woody |
| *Clerodendrum wallichii* Merr. | Lamiaceae | | Woody |
| *Clitoria ternatea* L. | Fabaceae | | Herb |
| *Clusia rosea* Jacq. | Clusiaceae | | Woody |
| *Cnicus benedictus* L. | Asteraceae | | Herb |
| *Coccoloba latifolia* Lam. | Polygonaceae | | Herb |
| *Cocculus hirsutus* (L.) W.Theob. | Menispermaceae | | Woody |
| *Codiaeum variegatum* (L.) A.Juss. var. variegatum | Euphorbiaceae | | Woody |
| *Coffea arabica* L. | Rubiaceae | | Woody |
| *Coix lacryma-jobi* L. | Poaceae | | Grass |
| *Coleus thyrsoideus* Baker | Lamiaceae | | Woody |
| *Colocasia esculenta* (L.) Schott | Araceae | | Herb |
| *Combretum celastroides* Welw. ex M.A.Lawson subsp. celastroides | Combretaceae | | Woody |
| *Combretum fruticosum* (Loefl.) Stuntz | Combretaceae | | Woody |
| *Combretum monticola* Engl. & Gilg | Combretaceae | | Woody |
| *Combretum racemosum* P.Beauv. | Combretaceae | | Woody |
| *Convolvulus arvensis* L. | Convolvulaceae | | Herb |
| *Conyza bonariensis* (L.) Cronq. | Asteraceae | | Herb |
| *Conyza canadensis* (L.) Cronq. | Asteraceae | | Herb |
| *Conyza primulifolia* (Lam.) Cuatrec. & Lourteig | Asteraceae | | Herb |
| *Conyza sumatrensis* (Retz.) E.Walker var. sumatrensis | Asteraceae | | Herb |
| *Conyza sumatrensis* (S.F.Blake) Pruski & G.Sancho, 2006 | Asteraceae | | Herb |
| *Corchorus olitorius* L. | Malvaceae | | Herb |
| *Corchorus tridens* L. | Malvaceae | | Herb |
| *Corchorus trilocularis* L. | Malvaceae | | Herb |
| *Cordia alliodora* (Ruiz & Pav.) Oken | Boraginaceae | | Woody |
| *Cordia myxa* L. | Boraginaceae | | Woody |
| *Cordia sebestena* L. | Boraginaceae | | Woody |
| *Coreopsis lanceolata* L. | Asteraceae | | Herb |
| *Coreopsis tinctoria* Nutt. | Asteraceae | | Herb |
| *Coriandrum sativum* L. | Apiaceae | | Herb |
| *Cornus capitata* Wall. | Cornaceae | | Woody |
| *Cornus florida* L. | Cornaceae | | Woody |
| *Cortaderia jubata* (Lemoine ex Carriere) Stapf | Poaceae | | Grass |
| *Cortaderia selloana* (Schult. & Schult.f.) Asch. & Graebn. | Poaceae | | Grass |
| *Corymbia citriodora* (Hook.) K.D.Hill & L.A.S.Johnson | Myrtaceae | | Woody |
| *Cosmos bipinnatus* Cav. | Asteraceae | | Herb |
| *Cosmos sulphureus* Cav. | Asteraceae | | Herb |
| *Costus afer* Ker Gawl. | Costaceae | | Herb |
| *Costus speciosus* (J.Koenig) Sm. | Costaceae | | Herb |
| *Cotoneaster coriaceus* Franch. | Rosaceae | | Woody |
| *Cotoneaster franchetii* Boiss. | Rosaceae | | Woody |
| *Cotoneaster glaucophyllus* Franch. | Rosaceae | | Woody |
| *Cotoneaster pannosus* Franch. | Rosaceae | | Woody |
| *Cotula sericea* L.fil. | Asteraceae | | Herb |
| *Crambe hispanica* L. | Brassicaceae | | Herb |
| *Crassula* hybrid | Acanthaceae | | Succulent |
| *Crassula nodulosa* var. nodulosa | Acanthaceae | | Succulent |
| *Crassula vaginata* subsp. minuta Toelken | Acanthaceae | | Succulent |
| *Crassula vaillantii* (Willd.) Roth | Crassulaceae | | Herb |
| *Crataegus* × *lavalleei* Herincq ex Lavallee | Rosaceae | | Woody |
| *Crataegus gracilior* J.B.Phipps | Rosaceae | | Woody |
| *Crataegus mexicana* Moç. & Sesse ex DC. | Rosaceae | | Woody |
| *Crataegus monogyna* Jacq. | Rosaceae | | Woody |
| *Crataegus phaenopyrum* (L.f.) Medik. | Rosaceae | | Woody |
| *Crataegus phaenopyrum* (L.fil.) Borkh. | Rosaceae | | Woody |
| *Crepis hypochaeridea* (DC.) Thell. | Asteraceae | | Herb |
| *Crescentia cujete* L. | Bignoniaceae | | Woody |
| *Crocosmia paniculata* | Iridaceae | | Bulb |
| *Crossandra infundibuliformis* (L.) Nees | Acanthaceae | | Woody |
| *Crossandra puberula* Klotzsch | Acanthaceae | | Woody |
| *Crotalaria agatiflora* Schweinf. | Fabaceae | | Woody |
| *Crotalaria agatiflora* subsp. agatiflora | Fabaceae | | Woody |
| *Crotalaria agatiflora* subsp. imperialis (Taub.) Polhill | Fabaceae | | Woody |
| *Crotalaria juncea* L. | Fabaceae | | Woody |
| Crotalaria lanceolata subsp. contigua Polhill | Fabaceae | | Woody |
| Croton bonplandianus Baill. | Euphorbiaceae | | Woody |
| Croton glandulosus L. | Euphorbiaceae | | Woody |
| Cryptomeria japonica (Thunb. ex L.f.) D.Don | Cupressaceae | | Woody |
| *Cryptostegia grandiflora* (Roxb.) R.Br. | Apocynaceae | | Woody |
| *Ctenanthe setosa* (Roscoe) Eichler | Marantaceae | | Herb |
| *Cucumis sativus* L. | Cucurbitaceae | | Herb |
| *Cunninghamia lanceolata* (Lamb.) Hook.f. | Cupressaceae | | Woody |
| *Cupressus arizonica* Greene | Cupressaceae | | Woody |
| *Cupressus arizonica* Greene var. arizonica | Cupressaceae | | Woody |
| *Cupressus arizonica* var. glabra (Sudw.) Little | Cupressaceae | | Woody |
| *Cupressus funebris* Endl. | Cupressaceae | | Woody |
| *Cupressus goveniana* Gordon | Cupressaceae | | Woody |
| *Cupressus lusitanica* Mill. | Cupressaceae | | Woody |
| *Cupressus lusitanica* Mill. var. lusitanica | Cupressaceae | | Woody |
| *Cupressus lusitanica* var. benthamii (Endl.) Carriere | Cupressaceae | | Woody |
| *Cupressus macrocarpa* Hartw. | Cupressaceae | | Woody |
| *Cupressus sempervirens* L. var. sempervirens | Cupressaceae | | Woody |
| Cupressus sempervirens var. horizontalis Gordon | Cupressaceae | | Woody |
| *Cupressus torulosa* D.Don | Cupressaceae | | Woody |
| *Cuscuta campestris* Yunck. | Convolvulaceae | | Herb |
| *Cuscuta suaveolens* Ser. | Convolvulaceae | | Herb |
| *Cyanthillium cinereum* (L.) H.Rob. | Asteraceae | | Herb |
| *Cyathea australis* (R.Br.) Domin | Cyatheaceae | | Fern |
| *Cyathea brownii* Domin | Cyatheaceae | | Fern |
| *Cyathea capensis* (L.f.) Sm. var. capensis | Cyatheaceae | | Woody |
| *Cyathea cooperi* (F.Muell.) Domin | Cyatheaceae | | Fern |
| *Cyclospermum leptophyllum* (Pers.) Sprague ex Britton & P.Wilson | Apiaceae | | Herb |
| *Cycnium tubulosum* subsp. montanum (N.E.Br.) O.J.Hansen | Scrophulariaceae | | Herb |
| *Cydonia oblonga* Mill. | Rosaceae | | Woody |
| *Cylindropuntia fulgida* (Engelm.) F.M.Knuth var. mamillata | Cactaceae | | Succulent |
| *Cylindropuntia imbricata* (Haw.) F.M.Knuth | Cactaceae | | Succulent |
| *Cylindropuntia imbricata* subsp. imbricata | Cactaceae | | Succulent |
| *Cylindropuntia imbricata* subsp. rosea (DC.) M.A.Baker | Cactaceae | | Succulent |
| *Cylindropuntia pallida* (Rose) F.M.Knuth | Cactaceae | | Succulent |
| *Cylindropuntia spinosior* (Engelm.) F.M.Knuth | Cactaceae | | Succulent |
| *Cymbalaria muralis* G.Gaertn., B.Mey. & Scherb. | Scrophulariaceae | | Herb |
| *Cynanchum* hybrid | Apocynaceae | | Fern |
| *Cynodon aethiopicus* Clayton & J.R.Harlan | Poaceae | | Grass |
| *Cynodon dactylon* (L.) Pers. | Poaceae | | Grass |
| *Cynodon decipiens* Caro & E.A.Sanchez | Poaceae | | Grass |
| *Cynodon nlemfuensis* Vanderyst | Poaceae | | Grass |
| *Cynoglossum amabile* Stapf & J.R.Drumm. | Boraginaceae | | Herb |
| *Cynoglossum coeruleum* A.DC. | Boraginaceae | | Herb |
| *Cynosurus echinatus* L. | Poaceae | | Grass |
| *Cyperus aureobrunneus* C.B.Clarke | Cyperaceae | | Grass |
| *Cyperus cyperoides* (L.) Kuntze | Cyperaceae | | Grass |
| *Cyperus rotundus* L. | Cyperaceae | | Grass |
| *Cyrtomium caryotideum* (Wall. ex Hook. & Grev.) C.Presl var. caryotideum | Dryopteridaceae | | Fern |
| *Cyrtomium falcatum* (L.fil.) C.Presl | Dryopteridaceae | | Herb |
| *Cystopteris fragilis* subsp. a auct. | Cystopteridaceae | | Herb |
| *Cytisus scoparius* (L.) Link 'Andreanus' | Fabaceae | | Woody |
| *Cytisus scoparius* subsp. scoparius | Fabaceae | | Woody |
| *Dacrydium cupressinum* Sol. ex Lamb. | Podocarpaceae | | Woody |
| *Dactylis glomerata* L. | Poaceae | | Grass |
| *Dactyloctenium aegyptium* (L.) Willd. | Poaceae | | Grass |
| *Dahlia imperialis* Roezl ex Ortgies | Asteraceae | | Woody |
| *Dalbergia sissoo* Roxb. ex DC. | Fabaceae | | Woody |
| *Dalechampia scandens* L. | Euphorbiaceae | | Woody |
| *Datura ferox* L. | Solanaceae | | Herb |
| *Datura innoxia* Mill. | Solanaceae | | Herb |
| *Datura metel* L. | Solanaceae | | Herb |
| *Datura stramonium* L. | Solanaceae | | Herb |
| *Daucus carota* L. | Solanaceae | | Herb |
| *Delonix regia* (Bojer ex Hook.) Raf. | Fabaceae | | Woody |
| *Delosperma pilosulum* L.Bolus | Mesembryanthemaceae | | Succulent |
| *Dendrocalamus latiflorus* Munro | Poaceae | | Woody |
| *Dendrocalamus strictus* (Roxb.) Nees | Poaceae | | Woody |
| *Deparia japonica* (Thunb.) M. Kato | Athyriaceae | | Herb |
| *Deschampsia caespitosa* (L.) P.Beauv. | Poaceae | | Grass |
| *Descurainia sophia* (L.) Webb ex Prantl | Brassicaceae | | Herb |
| *Desmanthus virgatus* (L.) Willd. | Fabaceae | | Woody |
| *Desmodium glutinosum* (Muhl. ex Willd.) Wood | Fabaceae | | Herb |
| *Desmodium incanum* (Sw.) DC. | Fabaceae | | Herb |
| *Desmodium tortuosum* (Sw.) DC. | Fabaceae | | Herb |
| *Desmodium uncinatum* (Jacq.) DC. | Fabaceae | | Herb |
| *Dianella caerulea* Sims | Asphodelaceae | | Herb |
| *Dichanthium aristatum* (Poir.) C.E.Hubb. | Poaceae | | Grass |
| *Dichondra micrantha* Urb. | Convolvulaceae | | Herb |
| *Dichondra repens* J.R.Forst. & G.Forst. | Convolvulaceae | | Herb |
| *Dichrocephala integrifolia* (L.f.) Kuntze subsp. integrifolia | Asteraceae | | Herb |
| *Dicksonia antarctica* Labill. | Dicksoniaceae | | Fern |
| *Digitalis purpurea* L. | Plantaginaceae | | Herb |
| *Digitaria ciliaris* (Retz.) Koeler | Poaceae | | Grass |
| *Digitaria didactyla* Willd. | Poaceae | | Grass |
| *Digitaria nuda* Schumach. | Poaceae | | Grass |
| *Digitaria sanguinalis* (L.) Scop. | Poaceae | | Grass |
| *Digitaria violascens* Link | Poaceae | | Grass |
| *Dioscorea diversifolia* Griseb. | Dioscoreaceae | | Herb |
| *Diplazium esculentum* (Retz.) Sw. | Woodsiaceae | | Fern |
| *Diplocyclos palmatus* (L.) C.Jeffrey | Cucurbitaceae | | Herb |
| *Diplotaxis muralis* (L.) DC. | Brassicaceae | | Herb |
| *Dolichandra unguis-cati* (L.) L.G.Lohmann | Bignoniaceae | | Herb |
| *Drymaria cordata* (L.) Willd. ex Schult. | Caryophyllaceae | | Herb |
| *Duchesnea indica* (Andrews) Focke | Rosaceae | | Herb |
| *Duranta erecta* L. | Verbenaceae | | Woody |
| *Dysphania ambrosioides* (L.) Mosyakin & Clemants | Chenopodiaceae | | Herb |
| *Dysphania carinata* (R.Br.) Mosyakin & Clemants | Chenopodiaceae | | Herb |
| *Dysphania cristata* (F.Muell.) Mosyakin & Clemants | Chenopodiaceae | | Herb |
| *Dysphania multifida* (L.) Mosyakin & Clemants | Chenopodiaceae | | Herb |
| *Dysphania pumilio* (R.Br.) Mosyakin & Clemants | Chenopodiaceae | | Herb |
| *Echinochloa colonum* (L.) Link | Poaceae | | Grass |
| *Echinochloa crus-galli* (L.) P.Beauv. | Poaceae | | Grass |
| *Echinopsis oxygona* (Link & Otto) Zucc. ex Pfeiff. | Cactaceae | | Succulent |
| *Echium plantagineum* L. | Boraginaceae | | Herb |
| *Echium vulgare* subsp. vulgare | Boraginaceae | | Herb |
| *Eclipta prostrata* (L.) L. | Asteraceae | | Herb |
| *Egeria densa* Planch. | Hydrocharitaceae | | Herb |
| *Eichhornia crassipes* (Mart.) Solms | Pontederiaceae | | Herb |
| *Elephantopus mollis* Kunth | Asteraceae | | Herb |
| *Eleusine africana* Kenn.-O'Byrne | Poaceae | | Herb |
| *Eleusine multiflora* Hochst. ex A.Rich. | Poaceae | | Grass |
| *Eleusine tristachya* (Lam.) Lam. | Poaceae | | Grass |
| *Eleutherine bulbosa* (Mill.) Urb. | Iridaceae | | Herb |
| *Elodea densa* (Planch.) Casp. | Hydrocharitaceae | | Herb |
| *Epipremnum aureum* (Linden & Andre) G.S.Bunting | Araceae | | Herb |
| *Eragrostis barrelieri* Daveau | Poaceae | | Grass |
| *Eragrostis pilosa* (L.) Beauv. | Poaceae | | Grass |
| *Eragrostis tef* (Zuccagni) Trotter | Poaceae | | Grass |
| *Eragrostis virescens* J.Presl | Poaceae | | Grass |
| *Erigeron bonariensis* L. | Asteraceae | | Herb |
| *Erigeron canadensis* L. | Asteraceae | | Woody |
| *Erigeron karvinskianus* DC. | Asteraceae | | Woody |
| *Erigeron primulifolius* (Lam.) Greuter | Asteraceae | | Herb |
| *Erigeron sumatrensis* Retz. | Asteraceae | | Herb |
| *Eriobotrya japonica* (Thunb. ) Lindl. | Rosaceae | | Woody |
| *Erodium cicutarium (L.)* L'Hér. | Geraniaceae | | Herb |
| *Erodium malacoides* (L.) L'Hér*.* | Geraniaceae | | Herb |
| *Erythrina crista-galli* L. | Fabaceae | | Woody |
| *Erythrostemon gilliesii* (Hook.) Klotzsch | Fabaceae | | Woody |
| *Eschscholzia californica* Cham. | Papaveraceae | | Herb |
| *Eucalyptus botryoides* Sm. | Myrtaceae | | Woody |
| *Eucalyptus camaldulensis* Dehnh. | Myrtaceae | | Woody |
| *Eucalyptus cinerea* F. Muell. ex Benth. | Myrtaceae | | Woody |
| *Eucalyptus cladocalyx* F.Muell. | Myrtaceae | | Woody |
| *Eucalyptus diversicolor* F. Muell. | Myrtaceae | | Woody |
| *Eucalyptus globulus* Labill. | Myrtaceae | | Woody |
| *Eucalyptus globulus* subsp. bicostata (Maiden, Blakely & Simmonds) J.B.Kirkp. | Myrtaceae | | Woody |
| *Eucalyptus grandis* W. Hill ex Maiden | Myrtaceae | | Woody |
| *Eucalyptus melliodora* A. Cunn. ex Schauer | Myrtaceae | | Woody |
| *Eucalyptus microcorys* F.Muell. | Myrtaceae | | Woody |
| *Eucalyptus regnans* F.Muell. | Myrtaceae | | Woody |
| *Eucalyptus robusta* Sm. | Myrtaceae | | Woody |
| *Eucalyptus sideroxylon* A.Cunn ex Woolls | Myrtaceae | | Woody |
| *Eucalyptus sideroxylon* subsp. sideroxylon | Myrtaceae | | Woody |
| *Euphorbia dentata* Michx. | Euphorbiaceae | | Herb |
| *Euphorbia helioscopia* L. | Euphorbiaceae | | Herb |
| *Euphorbia heterophylla* L. | Euphorbiaceae | | Herb |
| *Euphorbia heterophylla* var. cyathophora (Murray) Griseb. | Euphorbiaceae | | Herb |
| *Euphorbia hirsuta* L. | Euphorbiaceae | | Herb |
| *Euphorbia hirta* L. | Euphorbiaceae | | Herb |
| *Euphorbia hypericifolia* L. | Euphorbiaceae | | Herb |
| *Euphorbia indica* Lam. | Euphorbiaceae | | Herb |
| *Euphorbia leucocephala* Lotsy | Euphorbiaceae | | Herb |
| *Euphorbia marginata* Pursh | Euphorbiaceae | | Herb |
| *Euphorbia milii* Des Moul. | Euphorbiaceae | | Herb |
| *Euphorbia nutans* Lag. | Euphorbiaceae | | Herb |
| *Euphorbia peplus* L. | Euphorbiaceae | | Herb |
| *Euphorbia prostrata* Aiton | Euphorbiaceae | | Herb |
| *Euphorbia pulcherrima* Willd. ex Klotzsch | Euphorbiaceae | | Herb |
| *Euphorbia serpens* Kunth | Euphorbiaceae | | Herb |
| *Euphorbia terracina* L. | Euphorbiaceae | | Herb |
| *Euphorbia tithymaloides* L. | Euphorbiaceae | | Herb |
| *Euphorbia tithymaloides* subsp. smallii (Millsp.) V.W.Steinm. | Euphorbiaceae | | Herb |
| *Facelis retusa* (Lam.) Sch.Bip. | Asteraceae | | Herb |
| *Fagopyrum esculentum* Moench | Polygonaceae | | Herb |
| *Fallopia convolvulus* (L.) Holub | Polygonaceae | | Herb |
| *Fallopia sachalinensis* (F. Schmidt) Ronse Decr. | Polygonaceae | | Herb |
| *Festuca rubra* subsp. rubra | Poaceae | | Grass |
| *Ficus carica* L. | Moraceae | | Woody |
| *Fimbristylis complanata* (Retz.) Link | Poaceae | | Grass |
| *Flaveria bidentis* (L.) Kuntze | Asteraceae | | Herb |
| *Foeniculum vulgare* Mill. | Apiaceae | | Herb |
| *Fragaria vesca* L. | Rosaceae | | Herb |
| *Fraxinus americana* L. | Oleaceae | | Woody |
| *Fraxinus angustifolia* Vahl | Oleaceae | | Woody |
| *Fraxinus pennsylvanica/velutina* | Oleaceae | | Woody |
| *Fumaria muralis* Koch | Fumariaceae | | Herb |
| *Fumaria officinalis* L. | Fumariaceae | | Herb |
| *Furcraea foetida* (L.) Haw. | Asparagaceae | | Succulent |
| *Furcraea selloana* K.Koch | Asparagaceae | | Succulent |
| *Gaillardia pulchella* Foug. | Asteraceae | | Herb |
| *Galinsoga parviflora* Cav. | Asteraceae | | Herb |
| *Gamochaeta antillana* (Urb.) Anderb. | Asteraceae | | Herb |
| *Gamochaeta pensylvanica* (Willd.) Cabrera | Asteraceae | | Herb |
| *Gasteria obliqua* (Aiton) Duval | Asphodelaceae | | Herb |
| *Genista monspessulana* (L.) L.A.S.Johnson | Fabaceae | | Woody |
| *Geranium molle* L. | Geraniaceae | | Herb |
| *Geranium purpureum* Vill. | Geraniaceae | | Herb |
| *Gladiolus longicollis* subsp. platypetalus (Baker) Goldblatt & J.C.Manning | Iridaceae | | Herb |
| *Gladiolus sericeovillosus* subsp. calvatus (Baker) Goldblatt | Iridaceae | | Bulb |
| *Glandularia aristigera* (S.Moore) Tronc. | Verbenaceae | | Herb |
| *Glaucium corniculatum* (L.) Rudolph | Papaveraceae | | Herb |
| *Gleditsia triacanthos* L. | Fabaceae | | Woody |
| *Glyceria maxima* (Hartm.) Holmb. | Poaceae | | Grass |
| *Glycyrrhiza glabra* L. | Fabaceae | | Herb |
| *Gnaphalium polycaulon* Pers. | Asteraceae | | Herb |
| *Gomphrena celosioides* Mart. | Amaranthaceae | | Herb |
| *Gomphrena globosa* L. | Amaranthaceae | | Herb |
| *Grevillea banksii* R.Br. | Proteaceae | | Woody |
| *Grevillea robusta* A.Cunn. ex R.Br. | Proteaceae | | Woody |
| *Guilleminea densa* (Willd.) Moq. | Amaranthaceae | | Herb |
| *Guizotia abyssinica* (L.fil.) Cass. | Asteraceae | | Herb |
| *Gymnema sylvestre* (Retz.) R.Br. ex Sm. | Asclepiadaceae | | Woody |
| *Hakea salicifolia* (Vent.) B.L. Burtt | Proteaceae | | Woody |
| *Hakea sericea* Schrad. & J.C.Wendl. | Proteaceae | | Woody |
| *Handroanthus chrysotrichus* (Mart. ex DC.) Mattos | Bignoniaceae | | Woody |
| *Harrisia bonplandii* (J.Parm. ex Pfeiff.) Britton & Rose | Cactaceae | | Succulent |
| *Harrisia martinii* (Labour.) Britton | Cactaceae | | Succulent |
| *Harrisia pomanensis* (F.A.C.Weber ex K.Schum.) Britton & Rose | Cactaceae | | Succulent |
| *Harrisia tortuosa* (J. Forbes ex Otto & A. Dietr.) Britton & Rose | Cactaceae | | Succulent |
| *Hedera canariensis* Willd. | Araliaceae | | Herb |
| *Hedera helix* L. | Araliaceae | | Herb |
| *Hedychium coccineum* Buch.-Ham. ex Sm. | Zingiberaceae | | Herb |
| *Hedychium coronarium* J.Koenig | Zingiberaceae | | Herb |
| *Hedychium flavescens* Carey ex Roscoe | Zingiberaceae | | Herb |
| *Hedychium gardnerianum* Sheph. ex Ker Gawl. | Zingiberaceae | | Herb |
| *Heimia myrtifolia* Cham. & Schltdl. | Lythraceae | | Woody |
| *Helianthus annuus* L. | Asteraceae | | Herb |
| *Helianthus argophyllus* Torr. & A.Gray | Asteraceae | | Herb |
| *Helichrysum luteoalbum* (L.) Rchb. | Asteraceae | | Herb |
| *Heliotropium curassavicum* L. | Boraginaceae | | Herb |
| *Helminthotheca echioides* (L) Holub | Asteraceae | | Herb |
| *Heptapleurum actinophyllum* (Endl.) Lowry & G.M.Plunkett | Araliaceae | | Woody |
| *Heptapleurum arboricola* Hayata | Araliaceae | | Woody |
| *Heterocentron subtriplinervium* (Link & Otto) A. Braun & C. D. Bouche | Melastomataceae | | Herb |
| *Hibiscus mastersianus* Hiern | Malvaceae | | Herb |
| *Hibiscus mutabilis* L. | Malvaceae | | Herb |
| *Hibiscus sabdariffa* L. | Malvaceae | | Herb |
| *Hibiscus syriacus* L. | Malvaceae | | Herb |
| *Hibiscus trionum* L. | Malvaceae | | Herb |
| *Hirschfeldia incana* (L.) Lagr.-Foss. | Brassicaceae | | Herb |
| *Holcus lanatus* L. | Poaceae | | Grass |
| *Holosteum umbellatum* subsp. umbellatum | Caryophyllaceae | | Herb |
| *Homalanthus populifolius* Graham | Euphorbiaceae | | Woody |
| *Hordeum murinum* L. | Poaceae | | Grass |
| *Hordeum murinum* subsp. glaucum (Steud.) Tzvelev | Poaceae | | Grass |
| *Hordeum murinum* subsp. leporinum (Link) Arcang. | Poaceae | | Grass |
| *Hordeum stenostachys* Godr. | Poaceae | | Grass |
| *Houttuynia cordata* Thunb. | Saururaceae | | Herb |
| *Hydrilla verticillata* (L.f.) Royle | Hydrocharitaceae | | Herb |
| *Hydrocleys nymphoides* (Humb. & Bonpl. ex Willd.) Buchenau | Limnocharitaceae | | Herb |
| *Hylocereus undatus* (Haw.) Britt. & Rose | Cactaceae | | Succulent |
| *Hymenosporum flavum* (Hook.) F.Muell. | Pittosporaceae | | Woody |
| *Hypericum forrestii* (Chitt.) N.Robson | Hypericaceae | | Woody |
| *Hypericum hookerianum* Wight & Arn. | Hypericaceae | | Woody |
| *Hypericum perforatum* L. | Hypericaceae | | Woody |
| *Hypericum pseudohenryi* N.Robson | Hypericaceae | | Woody |
| *Hypochaeris glabra* var. capensis DC., 1837 | Asteraceae | | Herb |
| *Hypochaeris radicata* L. | Asteraceae | | Herb |
| *Hypoestes phyllostachya* Baker | Acanthaceae | | Herb |
| *Hypoestes sanguinolenta* L. | Acanthaceae | | Herb |
| *Hypoxis decumbens* L. | Hypoxidaceae | | Herb |
| *Hypoxis obtusa* Burch. ex Ker Gawl. | Hypoxidaceae | | Bulb |
| *Ibicella lutea* (Lindl.) Van Eselt. | Martyniaceae | | Herb |
| *Impatiens walleriana* Hook.fil. | Balsaminaceae | | Woody |
| *Imperata cylindrica* (L.) Raeusch. | Poaceae | | Grass |
| *Indigofera heterantha* Wall. ex Brandis | Fabaceae | | Woody |
| *Ipomoea alba* L. | Convolvulaceae | | Herb |
| *Ipomoea arachnosperma* Welw. | Convolvulaceae | | Herb |
| *Ipomoea carnea* Jacq. ssp. fistulosa (Mart. ex Choisy) D.F.Austin | Convolvulaceae | | Herb |
| *Ipomoea coccinea* L. | Convolvulaceae | | Herb |
| *Ipomoea gossypioides* Parodi | Convolvulaceae | | Herb |
| *Ipomoea hederifolia* L. | Convolvulaceae | | Herb |
| *Ipomoea indica* (Burm.) Merr. | Convolvulaceae | | Herb |
| *Ipomoea intrapilosa* Rose | Convolvulaceae | | Herb |
| *Ipomoea nil* (L.) Roth | Convolvulaceae | | Herb |
| *Ipomoea purpurea* (L.) Roth | Convolvulaceae | | Herb |
| *Iris pseudacorus* L. | Iridaceae | | Herb |
| *Ixora coccinea* L. | Rubiaceae | | Woody |
| *Jacaranda mimosifolia* D. Don | Bignoniaceae | | Woody |
| *Jatropha curcas* L. | Euphorbiaceae | | Woody |
| *Jatropha multifida* L. | Euphorbiaceae | | Woody |
| *Jatropha podagrica* Hook. | Euphorbiaceae | | Succulent |
| *Juncus bufonius* L. | Poaceae | | Grass |
| *Juncus capitatus* Weigel | Poaceae | | Grass |
| *Juncus sonderianus* Buchenau | Poaceae | | Grass |
| *Juncus tenuis* Willd. | Poaceae | | Grass |
| *Juniperus bermudiana* L. | Cupressaceae | | Woody |
| *Juniperus virginiana* L. | Cupressaceae | | Woody |
| *Kalanchoe beharensis* Drake | Crassulaceae | | Succulent |
| *Kalanchoe fedtschenkoi* Raym.-Hamet & H.Perrier | Crassulaceae | | Succulent |
| *Kalanchoe paniculata* Harv. | Crassulaceae | | Succulent |
| *Kalanchoe pinnata* (Lam.) Pers. | Crassulaceae | | Succulent |
| *Kalanchoe prolifera* (Bowie ex Hook.) Raym.-Hamet | Crassulaceae | | Succulent |
| *Kalanchoe tubiflora* (Harv.) Raym.-Hamet | Crassulaceae | | Succulent |
| *Koelreuteria paniculata* Laxm. | Sapindaceae | | Woody |
| *Lactuca indica* L. | Asteraceae | | Herb |
| *Lactuca serriola* L. | Asteraceae | | Herb |
| *Lagerstroemia indica* L. | Lythraceae | | Woody |
| *Lagerstroemia speciosa* (L.) Pers. | Lythraceae | | Woody |
| *Lamarckia aurea* (L.) Moench | Poaceae | | Grass |
| *Lamium amplexicaule* L. | Lamiaceae | | Herb |
| *Lamium galeobdolon* (L.) L. | Lamiaceae | | Herb |
| *Lantana camara* L. | Verbenaceae | | Woody |
| *Lantana camara* subsp. aculeata (L.) R.W.Sanders | Verbenaceae | | Woody |
| *Lantana montevidensis* (Spreng.) Briq. | Verbenaceae | | Woody |
| *Lantana trifolia* L. | Verbenaceae | | Woody |
| *Lappula heteracantha* (Ledeb.) Gürke | Boraginaceae | | Herb |
| *Lathyrus latifolius* L. | Fabaceae | | Herb |
| *Lepidium bonariense* L. | Brassicaceae | | Herb |
| *Lepidium didymum* L. | Brassicaceae | | Herb |
| *Lepidium draba* L. | Brassicaceae | | Herb |
| *Lepidium virginicum* L. | Brassicaceae | | Herb |
| *Leptospermum scoparium* Forst. | Myrtaceae | | Woody |
| *Lespedeza cuneata* (Dum.Cours.) G.Don | Fabaceae | | Woody |
| *Leucaena leucocephala* (Lam.) de Wit subsp. leucocephala | Fabaceae | | Woody |
| *Leucaena leucocephala* subsp. glabrata (Rose) Zarate | Fabaceae | | Woody |
| *Leucanthemum vulgare* Lam. | Asteraceae | | Herb |
| *Leucas lavandulifolia* Sm. | Lamiaceae | | Herb |
| *Ligustrum japonicum* Thunb. | Oleaceae | | Woody |
| *Ligustrum lucidum* W.T.Aiton | Oleaceae | | Woody |
| *Ligustrum ovalifolium* Hassk. | Oleaceae | | Woody |
| *Ligustrum sinense* Lour. | Oleaceae | | Woody |
| *Lilium formosanum* A.Wallace | Liliaceae | | Herb |
| *Linaria dalmatica* (L.) Mill. | Scrophulariaceae | | Herb |
| *Linaria genistifolia* (L.) Mill. | Scrophulariaceae | | Herb |
| *Linaria maroccana* Hook.fil. | Scrophulariaceae | | Herb |
| *Linaria vulgaris* Mill. | Scrophulariaceae | | Herb |
| *Litsea glutinosa* (Lour.) C.B. Robinson | Lauraceae | | Woody |
| *Livistona chinensis* (Jacq.) R.Br. ex Mart. | Arecaceae | | Woody |
| Lobularia maritima (L.) Desv. | Brassicaceae | | Herb |
| Lolium hybrid | Poaceae | | Grass |
| Lolium multiflorum Lam. | Poaceae | | Grass |
| Lolium perenne L. | Poaceae | | Grass |
| Lolium temulentum L. | Poaceae | | Grass |
| Lonicera japonica Thunb. var. Halliana | Caprifoliaceae | | Herb |
| *Lotus corniculatus* L. | Fabaceae | | Herb |
| *Ludwigia palustris* (L.) Elliott | Onagraceae | | Herb |
| *Lupinus angustifolius* L. | Fabaceae | | Herb |
| *Lycianthes rantonnetii* (CarriÃ¨re ex Lesc.) Bitter | Solanaceae | | Herb |
| *Lygodium japonicum* (Thunb.) Sw. | Lygodiaceae | | Herb |
| *Lysiloma latisiliquum* (L.) Benth. | Fabaceae | | Woody |
| *Lysimachia arvensis* subsp. arvensis | Primulaceae | | Herb |
| *Lysimachia ovalis* (Ruiz & Pav.) U.Manns & Anderb. | Primulaceae | | Herb |
| *Lythrum hyssopifolia* L. | Lythraceae | | Herb |
| *Macadamia integrifolia* Maiden & Betche | Proteaceae | | Woody |
| *Macroptilium atropurpureum* (DC.) Urb. | Fabaceae | | Herb |
| *Macrothelypteris torresiana* (Gaudich.) Ching | Thelypteridaceae | | Herb |
| *Mahonia oiwakensis* Hayata | Berberidaceae | | Woody |
| *Malva arborea* (L.) Webb & Berthel. | Malvaceae | | Herb |
| *Malva multiflora* (Cav.) Soldano, Banfi & Galasso | Malvaceae | | Herb |
| *Malva neglecta* Wallr. | Malvaceae | | Herb |
| *Malva parviflora* var. parviflora | Malvaceae | | Herb |
| *Malva pusilla* Sm. | Malvaceae | | Herb |
| *Malva sylvestris* L. | Malvaceae | | Herb |
| *Malva verticillata* L. var. verticillata | Malvaceae | | Herb |
| *Malva verticillata* var. crispa L. | Malvaceae | | Herb |
| *Malvastrum coromandelianum* (L.) Garcke | Malvaceae | | Herb |
| *Malvaviscus penduliflorus* DC. | Malvaceae | | Woody |
| *Mangifera indica* L. | Anacardiaceae | | Woody |
| *Manihot esculenta* Crantz | Euphorbiaceae | | Woody |
| *Manihot grahamii* Hook. | Euphorbiaceae | | Woody |
| *Maranta leuconeura* E.Morren | Marantaceae | | Herb |
| *Marchantia polymorpha* subsp. ruderalis Bischl. & Boissel.-Dub. | Marchantiaceae | | Herb |
| *Marrubium vulgare* L. | Lamiaceae | | Woody |
| *Medicago falcata* L. | Fabaceae | | Herb |
| *Medicago laciniata* (L.) Mill. | Fabaceae | | Herb |
| *Medicago lupulina* L. | Fabaceae | | Herb |
| *Medicago polymorpha* L. | Fabaceae | | Herb |
| *Medicago sativa* L. | Fabaceae | | Herb |
| *Megaskepasma erythrochlamys* Lindau | Acanthaceae | | Herb |
| *Melaleuca armillaris* subsp. armillaris | Myrtaceae | | Woody |
| *Melaleuca quinquenervia* (Cav.) S.T.Blake | Myrtaceae | | Woody |
| *Melaleuca styphelioides* Sm. | Myrtaceae | | Woody |
| *Melanthera scandens* (Schumach. & Thonn.) Roberty | Asteraceae | | Herb |
| *Melastoma malabathricum* L. | Melastomataceae | | Woody |
| *Melia azedarach* L. | Meliaceae | | Woody |
| *Melilotus alba* Medik. | Fabaceae | | Herb |
| *Melilotus indicus* (L.) All. | Fabaceae | | Herb |
| *Melilotus officinalis* (L.) Lam. | Fabaceae | | Herb |
| *Melinis repens* (Willd.) Zizka | Poaceae | | Grass |
| *Melinis repens* subsp. repens | Poaceae | | Grass |
| *Mentha pulegium* L. | Lamiaceae | | Herb |
| *Mesosphaerum pectinatum* (L.) Kuntze | Lamiaceae | | Herb |
| *Microlaena stipoides* (Labill.) R.Br. | Poaceae | | Grass |
| *Mimosa albida* Humb. & Bonpl. ex Willd. | Fabaceae | | Woody |
| *Mimosa pudica* L. | Fabaceae | | Woody |
| *Mimosa pudica* var. hispida Brenan | Fabaceae | | Woody |
| *Mirabilis jalapa* L. | Nyctaginaceae | | Herb |
| *Misopates orontium* subsp. orontium | Plantaginaceae | | Herb |
| *Modiola caroliniana* (L.) G.Don | Malvaceae | | Herb |
| *Momordica charantia* L. | Cucurbitaceae | | Herb |
| *Monstera deliciosa* Liebm. | Araceae | | Herb |
| *Montanoa bipinnatifida* (Kunth) K.Koch | Asteraceae | | Woody |
| *Montanoa hibiscifolia* (Benth.) K.Koch | Asteraceae | | Woody |
| *Moringa oleifera* Lam. | Moringaceae | | Woody |
| *Morus alba* L. | Moraceae | | Woody |
| *Morus alba* L. 'Laciniata' | Moraceae | | Woody |
| *Morus japonica* Audib. | Moraceae | | Woody |
| *Morus nigra* L. | Moraceae | | Woody |
| *Mucuna pruriens* var. utilis (Wall. ex Wight) Baker ex Burck | Fabaceae | | Herb |
| *Myoporum laetum* G.Forst. | Myoporaceae | | Woody |
| *Myosotis arvensis* (L.) Hill | Boraginaceae | | Herb |
| *Myosotis sylvatica* Ehrh. ex Hoffm. | Boraginaceae | | Herb |
| *Myriophyllum aquaticum* (Vell.) Verdc. | Haloragaceae | | Herb |
| *Myriophyllum spicatum* L. | Haloragaceae | | Herb |
| *Myrtillocactus geometrizans* (Mart. ex Pfeiff.) Console | Cactaceae | | Succulent |
| *Nandina domestica* Thunb. | Berberidaceae | | Woody |
| *Nassella neesiana* (Trin. & Rupr.) Barkworth | Poaceae | | Grass |
| *Nassella tenuissima* (Trin.) Barkworth | Poaceae | | Grass |
| *Nassella trichotoma* (Nees) Hack. & Arechav. | Poaceae | | Grass |
| *Nasturtium officinale* R.Br. | Brassicaceae | | Herb |
| *Nasturtium officinale* W.T.Aiton | Brassicaceae | | Herb |
| *Nephrolepis cordifolia* (L.) C.Presl | Nephrolepidaceae | | Herb |
| *Nephrolepis exaltata* (L.) Schott | Nephrolepidaceae | | Herb |
| *Nerium oleander* L. | Apocynaceae | | Woody |
| *Nicandra physalodes* (L.) Gaertn. | Solanaceae | | Herb |
| *Nicotiana glauca* Graham | Solanaceae | | Woody |
| *Nicotiana longiflora* Cav. | Solanaceae | | Woody |
| *Nicotiana tabacum* L. | Solanaceae | | Woody |
| *Nierembergia linariifolia* Graham | Solanaceae | | Herb |
| *Nierembergia linariifolia* var. glabriuscula (Dunal) A.A.Cocucci & Hunz. | Solanaceae | | Herb |
| *Nolina bigelovii* (Torr.) S.Watson | Asparagaceae | | Woody |
| *Nothoscordum gracile* (Aiton) Stearn | Amaryllidaceae | | Woody |
| *Nymphaea mexicana* Zucc. | Nymphaeaceae | | Herb |
| *Nymphaea* X *marliacea* Lat.-Marl. | Nymphaeaceae | | Herb |
| *Nymphoides peltata* (S. G. Gmel.) Kuntze | Menyanthaceae | | Herb |
| *Odontonema cuspidatum* (Nees) Kuntze | Acanthaceae | | Woody |
| *Odontonema strictum* (Nees) Kuntze | Acanthaceae | | Woody |
| *Oenothera affinis* Cambess. | Onagraceae | | Herb |
| *Oenothera biennis* L. | Onagraceae | | Herb |
| *Oenothera drummondii* subsp. drummondii | Onagraceae | | Herb |
| *Oenothera glazioviana* Micheli | Onagraceae | | Herb |
| *Oenothera indecora* Cambess. | Onagraceae | | Herb |
| *Oenothera jamesii* Torr. & A.Gray | Onagraceae | | Herb |
| *Oenothera laciniata* Hill | Onagraceae | | Herb |
| *Oenothera lindheimeri* (Engelm. & A.Gray) W.L.Wagner & Hoch | Onagraceae | | Herb |
| *Oenothera parodiana* Munz | Onagraceae | | Herb |
| *Oenothera rosea* L'Herit. ex Aiton | Onagraceae | | Herb |
| *Oenothera stricta* Ledeb. ex Link subsp. stricta | Onagraceae | | Herb |
| *Oenothera tetraptera* Cav. | Onagraceae | | Herb |
| *Oenothera villosa* Thunb. | Onagraceae | | Herb |
| *Olyra latifolia* L. | Poaceae | | Grass |
| *Opuntia aurantiaca* Gillies ex Lindl. | Cactaceae | | Succulent |
| *Opuntia cochenillifera* (L.) Mill. | Cactaceae | | Succulent |
| *Opuntia elata* Link & Otto ex Salm-Dyck var. elata | Cactaceae | | Succulent |
| *Opuntia engelmannii* Salm-Dyck ex Engelm. | Cactaceae | | Succulent |
| *Opuntia ficus-indica* (L.) Mill. | Cactaceae | | Succulent |
| *Opuntia humifusa* (Raf.) Raf. | Cactaceae | | Succulent |
| *Opuntia humifusa* subsp. minor (Engelm.) R.Crook & Mottram | Cactaceae | | Succulent |
| *Opuntia leucotricha* DC. | Cactaceae | | Succulent |
| *Opuntia megapotamica* Arechav. | Cactaceae | | Succulent |
| *Opuntia microdasys* (Lehm.) Pfeiff. | Cactaceae | | Succulent |
| *Opuntia monacantha* Haw. | Cactaceae | | Succulent |
| *Opuntia robusta* H.L. Wendl. | Cactaceae | | Succulent |
| *Opuntia robusta* J.C. Wendl. | Cactaceae | | Succulent |
| *Opuntia spinulifera* Salm-Dyck | Cactaceae | | Succulent |
| *Opuntia stricta* (Haw.) Haw. | Cactaceae | | Succulent |
| *Opuntia tomentosa* Salm-Dyck | Cactaceae | | Succulent |
| *Oxalis corniculata* L. | Oxalidaceae | | Herb |
| *Oxalis latifolia* Kunth | Oxalidaceae | | Herb |
| *Pachystachys lutea* Nees | Acanthaceae | | Woody |
| *Panicum miliaceum* L. | Poaceae | | Grass |
| *Panicum schinzii* Hack. | Poaceae | | Grass |
| *Papaver rhoeas* L. | Papaveraceae | | Herb |
| *Paraserianthes lophantha* (Willd.) I.C.Nielsen | Fabaceae | | Woody |
| *Paraserianthes lophantha* (Willd.) I.C.Nielsen subsp. lophantha | Fabaceae | | Woody |
| *Parkinsonia aculeata* L. | Fabaceae | | Woody |
| *Paronychia brasiliana* var. brasiliana | Caryophyllaceae | | Herb |
| *Paronychia brasiliana* var. pubescens Chaudhri | Caryophyllaceae | | Herb |
| *Parthenium hysterophorus* L. | Asteraceae | | Herb |
| *Parthenocissus quinquefolia* (L.) Planch. | Vitaceae | | Woody |
| *Paspalum dilatatum* Poir. | Poaceae | | Grass |
| *Paspalum distichum* L. | Poaceae | | Grass |
| *Paspalum notatum* Flugge | Poaceae | | Grass |
| *Paspalum quadrifarium* Lam. | Poaceae | | Grass |
| *Paspalum urvillei* Steud. | Poaceae | | Grass |
| *Passiflora caerulea* L. | Passifloraceae | | Herb |
| *Passiflora edulis* f. flavicarpa O.Deg. | Passifloraceae | | Herb |
| *Passiflora edulis* Sims | Passifloraceae | | Herb |
| *Passiflora foetida* L. | Passifloraceae | | Herb |
| *Passiflora suberosa* L. | Passifloraceae | | Herb |
| *Passiflora subpeltata* Ortega | Passifloraceae | | Herb |
| *Passiflora tarminiana* Coppens & V.E.Barney | Passifloraceae | | Herb |
| *Passiflora tripartita* var. mollissima (Kunth) Holm-Niels. & P.JÃ¸rg. | Passifloraceae | | Herb |
| *Pastinaca sativa* L. | Apiaceae | | Herb |
| *Paulownia tomentosa* (Thunb.) Steud. | Paulowniaceae | | Woody |
| *Peganum harmala* L. | Nitrariaceae | | Woody |
| *Peniocereus serpentinus* (Lag. & Rodr.) N.P.Taylor | Cactaceae | | Succulent |
| *Pennisetum clandestinum* Chiov. | Poaceae | | Grass |
| *Pennisetum purpureum* Schumach. | Poaceae | | Grass |
| *Pennisetum setaceum* (Forssk.) Chiov. | Poaceae | | Grass |
| *Pennisetum setaceum/villosum* | Poaceae | | Grass |
| *Pennisetum villosum* R. Br. ex Fresen. | Poaceae | | Grass |
| *Pereskia aculeata* Mill. | Cactaceae | | Herb |
| *Persea americana* Mill. | Lauraceae | | Woody |
| *Persicaria amphibia* (L.) Gray | Polygonaceae | | Herb |
| *Persicaria capitata* (Buch.-Ham. ex D.Don) | Polygonaceae | | Herb |
| *Persicaria hydropiper* (L.) Delarbre | Polygonaceae | | Herb |
| *Persicaria hydropiper* (L.) Spach | Polygonaceae | | Herb |
| *Persicaria lapathifolia* (L.) Delarbre | Polygonaceae | | Herb |
| *Persicaria lapathifolia* (L.) Gray | Polygonaceae | | Herb |
| *Persicaria lapathifolia* subsp. lapathifolia | Polygonaceae | | Herb |
| *Persicaria limbata* (Meisn.) H.Hara | Polygonaceae | | Herb |
| *Persicaria nepalensis* (Meisn.) H.Gross | Polygonaceae | | Herb |
| *Phalaris angusta* Nees ex Trin. | Poaceae | | Grass |
| *Phalaris aquatica* L. | Poaceae | | Grass |
| *Phalaris arundinacea* L. | Poaceae | | Grass |
| *Phalaris canariensis* L. | Poaceae | | Grass |
| *Phalaris minor* Retz. | Poaceae | | Grass |
| *Phlebodium aureum* (L.) J.Sm. | Polypodiaceae | | Herb |
| *Phoenix canariensis* H.Wildpret | Arecaceae | | Woody |
| *Phormium tenax* J.R.Forst. & G.Forst. | Phormiaceae | | Herb |
| *Phragmites australis* (Cav.) Trin. ex Steud. | Poaceae | | Grass |
| *Phragmites australis* subsp. australis | Poaceae | | Grass |
| *Phyla canescens* (Kunth) Greene | Verbenaceae | | Herb |
| *Phyla nodiflora* var. minor (Gillies & Hook.) N.O'Leary & Múlgura | Verbenaceae | | Woody |
| *Phyllanthus fraternus* G.L.Webster | Euphorbiaceae | | Herb |
| *Physalis angulata* L. | Solanaceae | | Herb |
| *Physalis peruviana* L. | Solanaceae | | Herb |
| *Physalis philadelphica* Lam. | Solanaceae | | Herb |
| *Physalis pubescens* L. | Solanaceae | | Herb |
| *Physalis viscosa* L. | Solanaceae | | Herb |
| *Phytolacca americana* L. | Phytolaccaceae | | Woody |
| *Phytolacca dioica* L. | Phytolaccaceae | | Woody |
| *Phytolacca octandra* L. | Phytolaccaceae | | Herb |
| *Picris hieracioides* L. | Asteraceae | | Herb |
| *Pilea microphylla* (L.) Liebm. | Urticaceae | | Succulent |
| *Pinus canariensis* C.Sm. | Pinaceae | | Woody |
| *Pinus elliottii* Engelm. var. elliottii | Pinaceae | | Woody |
| *Pinus elliottii* var. densa Little & K.W.Dorman | Pinaceae | | Woody |
| *Pinus engelmannii* Carrière | Pinaceae | | Woody |
| *Pinus halepensis* Mill. | Pinaceae | | Woody |
| *Pinus leiophylla* Schltdl. & Cham. | Pinaceae | | Woody |
| *Pinus luchuensis* Mayr | Pinaceae | | Woody |
| *Pinus massoniana* Lamb. | Pinaceae | | Woody |
| *Pinus montezumae* Lamb. var. montezumae | Pinaceae | | Woody |
| *Pinus nigra* subsp. laricio Maire | Pinaceae | | Woody |
| *Pinus oocarpa* Schiede | Pinaceae | | Woody |
| *Pinus patula* Schiede ex Schltdl. & Cham. | Pinaceae | | Woody |
| *Pinus patula* Schltdl. & Cham. var. patula | Pinaceae | | Woody |
| *Pinus patula* var. longipedunculata Loock ex Martinez | Pinaceae | | Woody |
| *Pinus patula* var. patula | Pinaceae | | Woody |
| *Pinus pinaster* Aiton | Pinaceae | | Woody |
| *Pinus pinea* L. | Pinaceae | | Woody |
| *Pinus pseudostrobus* Lindl. var. pseudostrobus | Pinaceae | | Woody |
| *Pinus radiata* D.Don | Pinaceae | | Woody |
| *Pinus roxburghii* Sarg. | Pinaceae | | Woody |
| *Pinus taeda* L. | Pinaceae | | Woody |
| *Pinus wallichiana* A.B.Jacks. | Pinaceae | | Woody |
| *Pistia stratiotes* L. | Araceae | | Herb |
| *Pittosporum undulatum* Vent. | Pittosporaceae | | Woody |
| *Pityrogramma calomelanos* (L.) Link | Pteridaceae | | Herb |
| *Plantago aristata* Michx. | Plantaginaceae | | Herb |
| *Plantago lanceolata* L. | Plantaginaceae | | Herb |
| *Plantago major* L. | Plantaginaceae | | Herb |
| *Plantago myosuros* Lam. | Plantaginaceae | | Herb |
| *Plantago rhodosperma* Decne. | Plantaginaceae | | Herb |
| *Plantago virginica* L. | Plantaginaceae | | Herb |
| *Platycerium bifurcatum* (Cav.) C.Chr. | Polypodiaceae | | Fern |
| *Plectranthus barbatus* var. grandis (L.H.Cramer) Lukhoba & A.J.Paton | Lamiaceae | | Woody |
| *Pleroma granulosum* (Desr.) D.Don | Melastomataceae | | Woody |
| *Pleroma urvilleanum* (DC.) P.J.F.Guim. & Michelang. | Melastomataceae | | Woody |
| *Poa annua* L. | Poaceae | | Grass |
| *Poa pratensis* L. | Poaceae | | Grass |
| *Poa trivialis* L. | Poaceae | | Grass |
| *Polycarpaea corymbosa* (L.) Lam. | Caryophyllaceae | | Herb |
| *Polycarpon tetraphyllum* (L.) L. | Caryophyllaceae | | Herb |
| *Polygala myrtifolia* var. myrtifolia | Polygalaceae | | Woody |
| *Polygonum aviculare* L. | Polygonaceae | | Herb |
| *Polypogon monspeliensis* (L.) Desf. | Poaceae | | Grass |
| *Polypogon viridis* (Gouan) Breistr. | Poaceae | | Grass |
| *Pombalia parviflora* (Mutis ex L.fil.) Paula-Souza | Violaceae | | Herb |
| *Pontederia cordata* L. | Pontederiaceae | | Herb |
| *Pontederia crassipes* Mart. | Pontederiaceae | | Herb |
| *Populus* × *canadensis* Moench | Salicaceae | | Woody |
| *Populus alba* L. | Salicaceae | | Woody |
| *Populus deltoides* Bartram ex Marshall subsp. deltoides | Salicaceae | | Woody |
| *Populus deltoides* Marshall | Salicaceae | | Woody |
| *Populus deltoides* subsp. deltoides | Salicaceae | | Woody |
| *Populus deltoides* W.Bartram ex Marshall | Salicaceae | | Woody |
| *Populus nigra* L. | Salicaceae | | Woody |
| *Populus nigra* L. var. italica M??nchh. | Salicaceae | | Woody |
| *Populus nigra* var. italica (Moench) Koehne | Salicaceae | | Woody |
| *Populus nigra* var. italica Munchh. | Salicaceae | | Woody |
| *Populus* x *canescens* (Aiton) Sm. | Salicaceae | | Woody |
| *Portulaca oleracea* L. | Portulacaceae | | Herb |
| *Portulaca pilosa* L. | Portulacaceae | | Herb |
| *Potentilla indica* (Andrews) Th.Wolf | Rosaceae | | Herb |
| *Primula malacoides* Franch. | Primulaceae | | Herb |
| *Neltuma glandulosa* Torr. var. glandulosa | Fabaceae | | Woody |
| *Neltuma glandulosa* Torr. var. torreyana (L.D.Benson) M.C.Johnst. | Fabaceae | | Woody |
| *Prosopis velutina* Wooton | Fabaceae | | Woody |
| *Prunella vulgaris* L. | Lamiaceae | | Herb |
| *Prunus armeniaca* L. | Rosaceae | | Woody |
| *Prunus cerasifera* Ehrh. | Rosaceae | | Woody |
| *Prunus persica* (L.) Batsch | Rosaceae | | Woody |
| *Prunus persica* (L.) Stokes | Rosaceae | | Woody |
| *Prunus serotina* Ehrh. | Rosaceae | | Woody |
| *Prunus serotina* var. salicifolia (Kunth) Koehne | Rosaceae | | Woody |
| *Psidium* × *durbanensis* | Myrtaceae | | Woody |
| *Psidium cattleianum* Afzel. ex Sabine | Myrtaceae | | Woody |
| *Psidium guajava* L. | Myrtaceae | | Woody |
| *Psidium guineense* Sw. | Myrtaceae | | Woody |
| *Pteris tremula* R.Br. | Pteridaceae | | Herb |
| *Pterocarya stenoptera* C.DC. | Juglandaceae | | Woody |
| *Puccinellia distans* (Jacq.) Parl. | Poaceae | | Grass |
| *Pueraria lobata* (Willd.) Ohwi | Fabaceae | | Herb |
| *Pueraria montana* var. lobata (Willd.) Maesen & S.M.Almeida ex Sanjappa & Predeep | Fabaceae | | Herb |
| *Pueraria montana* var. montana | Fabaceae | | Herb |
| *Punica granatum* L. | Lythraceae | | Woody |
| *Pyracantha angustifolia* (Franch.) C.K. Schneid. | Rosaceae | | Woody |
| *Pyracantha angustifolia*/*crenulata* | Rosaceae | | Woody |
| *Pyracantha coccinea* M.Roem. | Rosaceae | | Woody |
| *Pyracantha crenulata* (D.Don) M.Roem. | Rosaceae | | Woody |
| *Pyracantha koidzumii* (Hayata) Rehder | Rosaceae | | Woody |
| *Pyrostegia venusta* (Ker Gawl.) Miers | Bignoniaceae | | Woody |
| *Pyrus communis* L. | Rosaceae | | Woody |
| *Quercus acutissima* Carruth. | Fagaceae | | Woody |
| *Quercus palustris* Muenchh. | Fagaceae | | Woody |
| *Quercus robur* L. | Fagaceae | | Woody |
| *Quercus rugosa* | Fagaceae | | Woody |
| *Quercus suber* L. | Fagaceae | | Woody |
| *Ranunculus* cf. *rionii* Lagger | Ranunculaceae | | Herb |
| *Ranunculus muricatus* L. | Ranunculaceae | | Herb |
| *Raphanus raphanistrum* L. | Brassicaceae | | Herb |
| *Raphanus sativus* L. | Brassicaceae | | Herb |
| *Rapistrum rugosum* (L.) All. | Brassicaceae | | Herb |
| *Reseda lutea* subsp. lutea | Resedaceae | | Herb |
| *Reynoutria* × *bohemica* Chrtek | Polygonaceae | | Herb |
| *Reynoutria sachalinensis* (F.Schmidt) Nakai | Polygonaceae | | Woody |
| *Rhaphiolepis indica* (L.) Lindl. | Rosaceae | | Woody |
| *Rhododendron indicum* (L.) Sweet | Ericaceae | | Woody |
| *Richardia brasiliensis* Gomes | Rubiaceae | | Herb |
| *Richardia humistrata* (Cham. & Schltdl.) Steud. | Rubiaceae | | Herb |
| *Richardia scabra* L. | Rubiaceae | | Herb |
| *Ricinus communis* L. var. communis | Euphorbiaceae | | Woody |
| *Rivina humilis* L. | Phytolaccaceae | | Herb |
| *Robinia pseudoacacia* L. | Fabaceae | | Woody |
| *Roldana petasitis* (Sims) H.Rob. & Brettell | Asteraceae | | Woody |
| *Rosa canina* L.er | Rosaceae | | Woody |
| *Rosa multiflora* Thunb. | Rosaceae | | Woody |
| *Rosa rubiginosa* L. | Rosaceae | | Woody |
| *Rosa* x *odorata* (Andr.) Sweet | Rosaceae | | Woody |
| *Rottboellia cochinchinensis* (Lour.) Clayton | Poaceae | | Grass |
| *Rubus affinis* Weihe | Rosaceae | | Woody |
| *Rubus armeniacus* Focke | Rosaceae | | Woody |
| *Rubus cuneifolius* Pursh | Rosaceae | | Woody |
| *Rubus cuneifolius* x R. rigidus? | Rosaceae | | Woody |
| *Rubus ellipticus* Sm. | Rosaceae | | Woody |
| *Rubus flagellaris* Willd. | Rosaceae | | Woody |
| *Rubus fruticosus* L. agg. | Rosaceae | | Woody |
| *Rubus fruticosus* x R. rigidus? | Rosaceae | | Woody |
| *Rubus immixtus* Gust. | Rosaceae | | Woody |
| *Rubus niveus* Thunb. | Rosaceae | | Woody |
| *Rubus pascuus* L.H.Bailey | Rosaceae | | Woody |
| *Rubus phoenicolasius* Maxim. | Rosaceae | | Woody |
| *Rubus rosifolius* Sm. | Rosaceae | | Woody |
| *Rubus ulmifolius* Schott | Rosaceae | | Woody |
| *Ruellia graecizans* Backer | Acanthaceae | | Woody |
| *Ruellia simplex* C.Wright | Acanthaceae | | Herb |
| *Ruellia tuberosa* L. | Acanthaceae | | Herb |
| *Rumex acetosella* subsp. acetosella | Polygonaceae | | Herb |
| *Rumex acetosella* subsp. angiocarpus (Murb.) Murb. | Polygonaceae | | Herb |
| Rumex brownii Campd. | Polygonaceae | | Herb |
| Rumex crispus L. | Polygonaceae | | Herb |
| *Rumex nepalensis* Spreng. | Polygonaceae | | Herb |
| *Rumex obtusifolius* subsp. obtusifolius | Polygonaceae | | Herb |
| Rumex usambarensis (Engl. ex Dammer) Dammer | Polygonaceae | | Herb |
| *Ruta graveolens* L. | Rutaceae | | Woody |
| *Saccharum officinarum* L. | Poaceae | | Herb |
| *Sagina procumbens* L. | Caryophyllaceae | | Herb |
| *Sagittaria latifolia* Willd. | Alismataceae | | Herb |
| *Sagittaria platyphylla* (Engelm.) J.G.Sm. | Alismataceae | | Herb |
| *Salix babylonica* L. var. babylonica | Salicaceae | | Woody |
| *Salix caprea* L. | Salicaceae | | Woody |
| *Salix fragilis* L. | Salicaceae | | Woody |
| *Salsola australis* R.Br. | Amaranthaceae | | Herb |
| *Salsola kali* L. | Amaranthaceae | | Herb |
| *Salsola tragus* L. | Amaranthaceae | | Herb |
| *Salvia coccinea* Buc'hoz ex Etl. | Lamiaceae | | Woody |
| *Salvia reflexa* Hornem. | Lamiaceae | | Herb |
| *Salvia sclarea* L. | Lamiaceae | | Herb |
| *Salvia tiliifolia* Vahl | Lamiaceae | | Herb |
| *Salvia verbenaca* L. | Lamiaceae | | Herb |
| *Salvinia molesta* D.S.Mitch. | Salviniaceae | | Herb |
| *Sambucus canadensis* L. | Adoxaceae | | Woody |
| *Sambucus nigra* L. | Adoxaceae | | Woody |
| *Sanchezia oblonga* Ruiz & Pav. | Acanthaceae | | Woody |
| *Schefflera actinophylla* (Endl.) Harms | Araliaceae | | Woody |
| *Schefflera arboricola* (Hayata) Merr. | Araliaceae | | Woody |
| *Schinus molle* L. | Anacardiaceae | | Woody |
| *Schinus terebinthifolia* Raddi | Anacardiaceae | | Woody |
| *Schkuhria pinnata* (Lam.) Kuntze | Asteraceae | | Herb |
| *Schoenoplectus tabernaemontani* (C.C.Gmel.) Palla | Cyperaceae | | Herb |
| *Schoenoplectus triqueter* (L.) Palla | Cyperaceae | | Herb |
| *Scleranthus annuus* L. | Caryophyllaceae | | Herb |
| *Scoparia dulcis* L. | Plantaginaceae | | Herb |
| *Scutellaria racemosa* Pers. | Lamiaceae | | Herb |
| *Selenicereus undatus* (Haw.) D.R.Hunt | Cactaceae | | Succulent |
| *Senna bicapsularis* (L.) Roxb. | Fabaceae | | Woody |
| *Senna corymbosa* (Lam.) H.S.Irwin & Barneby | Fabaceae | | Woody |
| *Senna didymobotrya* (Fresen.) H.S.Irwin & Barneby | Fabaceae | | Woody |
| *Senna hirsuta* (L.) H.S.Irwin & Barneby | Fabaceae | | Woody |
| *Senna multiglandulosa* (Jacq.) H.S.Irwin & Barneby | Fabaceae | | Woody |
| *Senna obtusifolia* (L.) H.S.Irwin & Barneby | Fabaceae | | Woody |
| *Senna occidentalis* (L.) Link | Fabaceae | | Woody |
| *Senna pendula* (Humb. & Bonpl. ex Willd.) H.S.Irwin & Barneby | Fabaceae | | Woody |
| *Senna septemtrionalis* (Viv.) H.S.Irwin & Barneby | Fabaceae | | Woody |
| *Sesbania bispinosa* (Jacq.) W. Wight var. bispinosa | Fabaceae | | Woody |
| *Sesbania punicea* (Cav.) Benth. | Fabaceae | | Woody |
| *Setaria italica* (L.) P.Beauv. | Poaceae | | Grass |
| *Setaria megaphylla* (Steud.) T.Durand & Schinz | Poaceae | | Grass |
| *Setaria pumila* (Poir.) Roem. & Schult. | Poaceae | | Grass |
| *Setaria verticillata* (L.) P.Beauv. | Poaceae | | Grass |
| *Sherardia arvensis* L. | Rubiaceae | | Woody |
| *Sigesbeckia orientalis* L. | Asteraceae | | Herb |
| *Silene dioica* (L.) Clairv. | Caryophyllaceae | | Herb |
| *Silene vulgaris* subsp. macrocarpa Turrill | Caryophyllaceae | | Herb |
| *Silybum marianum* (L.) Gaertn. | Asteraceae | | Herb |
| *Sinapis arvensis* L. | Brassicaceae | | Herb |
| *Sisymbrium irio* L. | Brassicaceae | | Herb |
| *Sisymbrium officinale* (L.) Scop. | Brassicaceae | | Herb |
| *Sisymbrium orientale* L. | Brassicaceae | | Herb |
| *Sisyrinchium micranthum* Cav. | Brassicaceae | | Herb |
| *Solanum aculeatissimum* Jacq. | Solanaceae | | Woody |
| *Solanum americanum* Mill. | Solanaceae | | Woody |
| *Solanum betaceum* Cav. | Solanaceae | | Woody |
| *Solanum capsicoides* All. | Solanaceae | | Woody |
| *Solanum chenopodioides* Lam. | Solanaceae | | Woody |
| *Solanum chrysotrichum* Schltdl. | Solanaceae | | Woody |
| *Solanum elaeagnifolium* Cav. | Solanaceae | | Herb |
| *Solanum lycopersicum* L. | Solanaceae | | Woody |
| *Solanum lycopersicum* var. cerasiforme (Alef.) Voss | Solanaceae | | Woody |
| *Solanum mauritianum* Scop. | Solanaceae | | Woody |
| *Solanum muricatum* Aiton | Solanaceae | | Woody |
| *Solanum nigrum* L. | Solanaceae | | Herb |
| *Solanum nigrum* L. sensu lato | Solanaceae | | Herb |
| *Solanum pseudocapsicum* L. | Solanaceae | | Herb |
| *Solanum retroflexum* Dun. | Solanaceae | | Woody |
| *Solanum sarrachoides* Sendtn. | Solanaceae | | Woody |
| *Solanum seaforthianum* Andrews | Solanaceae | | Herb |
| *Solanum sisymbriifolium* Lam. | Solanaceae | | Woody |
| *Solanum torvum* Sw. | Solanaceae | | Woody |
| *Solanum triflorum* Nutt. | Solanaceae | | Woody |
| *Solanum tuberosum* L. | Solanaceae | | Woody |
| *Solanum viarum* Dunal | Solanaceae | | Woody |
| *Solidago altissima* L. | Asteraceae | | Herb |
| *Solidago gigantea* Aiton | Asteraceae | | Herb |
| *Sonchus asper* (L.) Hill | Asteraceae | | Herb |
| *Sonchus oleraceus* L. | Asteraceae | | Herb |
| *Sorghum halepense* (L.) Pers. | Poaceae | | Grass |
| *Spartium junceum* L. | Fabaceae | | Woody |
| *Spathodea campanulata* Beauv. | Bignoniaceae | | Woody |
| *Spergula arvensis* L. | Caryophyllaceae | | Herb |
| *Spergularia bocconei* (Scheele) Graebn. | Caryophyllaceae | | Herb |
| *Spergularia media* (L.) C.Presl | Caryophyllaceae | | Herb |
| *Sphaeralcea bonariensis* (Cav.) Griseb. | Malvaceae | | Herb |
| *Sphaeropteris cooperi* (Hook. ex F.Muell.) R.M.Tryon | Cyatheaceae | | Fern |
| *Sphagneticola trilobata* (L.) Pruski | Asteraceae | | Herb |
| *Sporobolus africanus* (Poir.) Robyns & Tournay | Poaceae | | Grass |
| *Stachytarpheta cayennensis* (Rich.) Vahl | Verbenaceae | | Herb |
| *Stachytarpheta mutabilis* (Jacq.) Vahl | Verbenaceae | | Herb |
| *Stachytarpheta urticifolia* Sims | Verbenaceae | | Herb |
| *Steinchisma hians* (Elliott) Nash | Poaceae | | Grass |
| *Stellaria apetala* Bernardino | Caryophyllaceae | | Herb |
| *Stellaria media* (L.) Vill. | Caryophyllaceae | | Herb |
| *Stellaria sennii* Chiov. | Caryophyllaceae | | Herb |
| *Stenotaphrum secundatum* (Walter) Kuntze | Poaceae | | Grass |
| *Strobilanthes isophyllus* (Nees) T.Anderson | Acanthaceae | | Woody |
| *Styphnolobium japonicum* (L.) Schott | Fabaceae | | Woody |
| *Symphyotrichum squamatum* (Spreng.) G.L.Nesom | Fabaceae | | Herb |
| *Symphyotrichum subulatum* (Michx.) G.L.Nesom | Fabaceae | | Herb |
| *Syncarpia glomulifera* (Sm.) Nied. | Myrtaceae | | Woody |
| *Syngonium podophyllum* Schott | Araceae | | Herb |
| *Syzygium australe* (J.C.Wendl. ex Link) B.Hyland | Myrtaceae | | Woody |
| *Syzygium cumini* (L.) Skeels | Myrtaceae | | Woody |
| *Syzygium jambos* (L.) Alston | Myrtaceae | | Woody |
| *Syzygium paniculatum* Gaertn. | Myrtaceae | | Woody |
| *Tagetes erecta* L. | Asteraceae | | Herb |
| *Tagetes minuta* L. | Asteraceae | | Herb |
| *Talinum paniculatum* (Jacq.) Gaertn. | Portulacaceae | | Herb |
| *Tamarindus indica* L. | Fabaceae | | Woody |
| *Tamarix chinensis* Lour. | Tamaricaceae | | Woody |
| *Tamarix ramosissima* Ledeb. | Tamaricaceae | | Woody |
| *Taraxacum bessarabicum* (Hornem.) Hand.-Mazz. | Asteraceae | | Herb |
| *Taraxacum brachyglossum* (Dahlst.) Dahlst. | Asteraceae | | Herb |
| *Taraxacum breviscapum* A.J.Richards | Asteraceae | | Herb |
| *Taraxacum brunneum* Soest | Asteraceae | | Herb |
| *Taraxacum disseminatum* G.E.Haglund | Asteraceae | | Herb |
| *Taraxacum ekmanii* Dahlst. | Asteraceae | | Herb |
| *Taraxacum hamatiforme* Dahlst. | Asteraceae | | Herb |
| *Taraxacum marklundii* Palmgr. | Asteraceae | | Herb |
| *Taraxacum officinale* Weber | Asteraceae | | Herb |
| *Taraxacum parvilobum* Dahlst. | Asteraceae | | Herb |
| *Taraxacum privum* Dahlst. | Asteraceae | | Herb |
| *Taraxacum serotinum* (Waldst. & Kit.) Poir. | Asteraceae | | Herb |
| *Taxodium distichum* (L.) Rich. | Cupressaceae | | Woody |
| *Tecoma stans* (L.) Juss. ex Kunth | Bignoniaceae | | Woody |
| *Tecoma stans* var. velutina DC. | Bignoniaceae | | Woody |
| *Telopea speciosissima* (Sm.) R.Br. | Proteaceae | | Woody |
| *Tephrocactus articulatus* (Pfeiff. ex Otto) Backeb. | Cactaceae | | Succulent |
| *Tephrosia purpurea* subsp. purpurea | Fabaceae | | Woody |
| *Thunbergia erecta* (Benth.) T.Anderson | Acanthaceae | | Woody |
| *Thunbergia grandiflora* (Roxb. ex Rottler) Roxb. | Acanthaceae | | Woody |
| *Thunbergia* hybrid | Acanthaceae | | Herb |
| *Tibouchina elegans* (Gardner) Cogn. | Melastomataceae | | Woody |
| *Tibouchina granulosa* (Desr.) Cogn. | Melastomataceae | | Woody |
| *Tibouchina mutabilis* (Vell.) Cogn. | Melastomataceae | | Woody |
| *Tibouchina urvilleana* (DC.) Cogn. | Melastomataceae | | Woody |
| *Tillandsia usneoides* (L.) L. | Bromeliaceae | | Herb |
| *Tipuana tipu (*Benth.) Kuntze | Fabaceae | | Woody |
| *Tithonia diversifolia* (Hemsl.) A.Gray | Asteraceae | | Woody |
| *Tithonia rotundifolia* (Mill.) S.F.Blake | Asteraceae | | Woody |
| *Toona ciliata* M.Roem. | Meliaceae | | Woody |
| *Torilis arvensis* (Huds.) Link. | Apiaceae | | Herb |
| *Torilis nodosa* (L.) Gaertn. | Apiaceae | | Herb |
| *Toxicodendron succedaneum* (L.) Kuntze | Picrodendraceae | | Woody |
| *Tradescantia fluminensis* Vell. | Commelinaceae | | Herb |
| *Tradescantia pallida* (Rose) D.R.Hunt | Commelinaceae | | Herb |
| *Tradescantia spathacea* Sw. | Commelinaceae | | Herb |
| *Tradescantia zebrina* Bosse | Commelinaceae | | Herb |
| *Tragopogon dubius* Scop. | Asteraceae | | Herb |
| *Tragopogon porrifolius* L. | Asteraceae | | Herb |
| *Tragus berteronianus* Schult. | Poaceae | | Grass |
| *Trichocereus spachianus* (Lem.) Riccob. | Cactaceae | | Succulent |
| *Tridax procumbens* L. | Asteraceae | | Herb |
| *Trifolium angustifolium* L. | Fabaceae | | Herb |
| *Trifolium cernuum* Brot. | Fabaceae | | Herb |
| *Trifolium dubium* Sibth. | Fabaceae | | Herb |
| *Trifolium hybridum* subsp. hybridum | Fabaceae | | Herb |
| *Trifolium pratense* var. pratense | Fabaceae | | Herb |
| *Trifolium repens* L. | Fabaceae | | Herb |
| *Trigonella foenum-graecum* L. | Fabaceae | | Herb |
| *Trigonella glabra* Thunb. | Fabaceae | | Herb |
| *Triplaris americana* L. | Polygonaceae | | Woody |
| *Tropaeolum majus* L. | Tropaeolaceae | | Herb |
| *Turritis glabra* L. | Brassicaceae | | Herb |
| *Typha capensis* (Rohrb.) N.E.Br. | Typhaceae | | Herb |
| *Typha domingensis* Pers. | Poaceae | | Grass |
| *Ulex europaeus* L. | Fabaceae | | Woody |
| *Ulmus minor* Mill. | Ulmaceae | | Woody |
| *Ulmus minor* subsp. procera (Salisb.) Franco | Ulmaceae | | Woody |
| *Ulmus parvifolia* Jacq. | Ulmaceae | | Woody |
| *Urena lobata* L. | Malvaceae | | Woody |
| *Urochloa panicoides* P.Beauv. | Poaceae | | Grass |
| *Urospermum picroides* (L.) Scop. ex F.W.Schmidt | Asteraceae | | Herb |
| *Urtica dioica* L. | Urticaceae | | Herb |
| *Urtica urens* L. | Urticaceae | | Herb |
| *Vachellia farnesiana* var. farnesiana | Scrophulariaceae | | Woody |
| *Verbascum thapsus* L. | Scrophulariaceae | | Herb |
| *Verbascum virgatum* Stokes | Scrophulariaceae | | Herb |
| *Verbena aristigera* S.Moore | Verbenaceae | | Herb |
| *Verbena bonariensis* L. | Verbenaceae | | Herb |
| *Verbena brasiliensis* Vell. | Verbenaceae | | Herb |
| *Verbena incompta* P.W.Michael | Verbenaceae | | Herb |
| *Verbena litoralis* Kunth | Verbenaceae | | Herb |
| *Verbena officinalis* L. | Verbenaceae | | Herb |
| *Verbena rigida* Spreng. | Verbenaceae | | Herb |
| Verbesina encelioides (Cav.) Benth. & Hook.f. ex A.Gray | Asteraceae | | Herb |
| *Vernicia fordii* (Hemsl.) Airy Shaw | Euphorbiaceae | | Woody |
| *Vernicia montana* Lour. | Euphorbiaceae | | Woody |
| *Veronica agrestis* L. | Plantaginaceae | | Herb |
| *Veronica hederifolia* L. | Plantaginaceae | | Herb |
| *Veronica persica* Poir. | Plantaginaceae | | Herb |
| *Veronica serpyllifolia* L. | Plantaginaceae | | Herb |
| *Viburnum odoratissimum* Ker Gawl. | Adoxaceae | | Woody |
| *Viburnum opulus* L. | Adoxaceae | | Woody |
| *Viburnum sinensis* Zeyh. ex Colla | Adoxaceae | | Woody |
| *Viburnum tinus* L. | Adoxaceae | | Woody |
| *Vicia benghalensis* L. | Fabaceae | | Herb |
| *Vicia hirsuta* (L.) Gray | Fabaceae | | Herb |
| *Vicia sativa* subsp. nigra Ehrh. | Fabaceae | | Herb |
| *Vicia sativa* subsp. sativa | Fabaceae | | Herb |
| *Vicia villosa* subsp. villosa | Fabaceae | | Herb |
| *Vinca major* L. | Apocynaceae | | Herb |
| *Viola arvensis* Murray | Violaceae | | Herb |
| *Viola hederacea* Labill. | Violaceae | | Herb |
| *Viola tricolor* L. | Lamiaceae | | Herb |
| *Vitex trifolia* L. | Lamiaceae | | Woody |
| *Vitex trifolia* var. heterophylla (Makino) Moldenke | Lamiaceae | | Woody |
| *Washingtonia robusta* H.Wendl. | Arecaceae | | Woody |
| *Wigandia urens* (Ruiz & Pav.) Kunth | Boraginaceae | | Woody |
| *Xanthium spinosum* L. | Asteraceae | | Herb |
| *Xanthium strumarium* L. | Asteraceae | | Herb |
| *Youngia japonica* (L.) DC. | Asteraceae | | Herb |
| *Yucca aloifolia* L. | Asparagaceae | | Woody |
| *Zannichellia palustris* subsp. pedicellata (Rosén & Wahlenb.) Arcang. | Potamogetonaceae | | Herb |
| *Zea mays* L. | Poaceae | | Herb |
| *Zinnia peruviana* (L.) L. | Asteraceae | | Herb |
| *Ziziphus mauritiana* Lam. | Rhamnaceae | | Woody |
